# Organoid‐based two‐step drug screening for rapid identification of chemotherapy‐resistant oesophageal squamous cell carcinoma and alternative therapies

**DOI:** 10.1002/ctm2.70534

**Published:** 2025-11-20

**Authors:** Chen‐Ye Shao, Sheng Ju, Xin Tong, Kang Hu, Yu Li, Yi‐xian Zhu, Jian Yang, Chang Li, Yu‐feng Xie, Yuan Cui, Wei‐jun Deng, Cheng Ding, Song‐bing Qin, Jun Zhao

**Affiliations:** ^1^ Department of Thoracic Surgery The First Affiliated Hospital of Soochow University Suzhou China; ^2^ Department of Thoracic Surgery, Institute of Minimally Invasive Thoracic Cancer Therapy and Translational Research The First Affiliated Hospital of Soochow University Suzhou China; ^3^ Department of Radiation Oncology The First Affiliated Hospital of Soochow University Suzhou China

**Keywords:** drug screening, drug sensitivity, oesophageal squamous cell carcinoma, precision medicine, tumour organoids

## Abstract

**Background:**

Despite guideline‐directed therapies, most patients with advanced oesophageal squamous cell carcinoma (ESCC) derive limited benefit and are unable to tolerate iterative treatment modifications. Therefore, timely identification of resistant cases and the provision of alternative therapeutic options are urgently needed.

**Methods:**

A large‐scale patient‐derived organoid model was established from locally advanced patients with ESCC who underwent perioperative chemotherapy and was validated for consistency with the parental tumours through histopathological, genomic and transcriptomic analysis. A novel two‐step drug screening method based on growth rate inhibition (GR) was developed, and drug sensitivity results were compared with clinical outcomes. Additionally, in vivo assays were conducted to evaluate alternative therapies using the *C*_max_/GR100 index.

**Results:**

ESCC organoids demonstrated high consistency with parental tumours in histopathology, genomics and transcriptomics. The two‐step drug screening method revealed a strong correlation with clinical responses (sensitivity 80%, specificity 85.7%, overall accuracy 83.3%) and significantly shortened the experimental timeline compared with the traditional drug screening method (23.08 ± 2.42 days vs. 45.75 ± 7.19 days, *p* < .001). Furthermore, we proposed a novel drug selection strategy based on *C*_max_/GR100 values, which provides a theoretical foundation for drug repurposing and offers alternative treatment options for patients resistant to TP (taxol + cisplatin) therapy, thereby facilitating precise re‐treatment in drug‐resistant subgroups.

**Conclusions:**

This method exhibits strong clinical applicability, supporting more accurate and timely decision‐making in ESCC management. The *C*_max_/GR100‐based drug screening strategy can dynamically identify alternative sensitive drugs for patients who fail first‐line therapies, enabling precise re‐treatment. This approach provides a valuable tool for translating laboratory findings into clinical practice.

## Graphical abstract

Organoid‐based two‐step drug screening method can dynamically identify alternative sensitive drugs for resistant patients, providing theoretical support for the ‘repositioning’ of these drugs. This method addresses a critical unmet need for patients refractory to first‐line therapies by enabling timely identification of effective treatment alternatives.

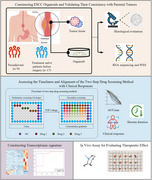

## 1 INTRODUCTION

Oesophageal carcinoma (EC), a neoplastic disorder arising from oesophageal mucosal epithelium, represents a highly prevalent and fatal disease of the digestive tract on a global scale. According to the 2022 Global Cancer Statistics Report,^1^ EC occupies the eleventh position in global cancer incidence rates while ranking seventh in cancer‐associated fatalities. EC has two major pathological subtypes with distinct geographical distributions: oesophageal adenocarcinoma (EAC) demonstrate higher prevalence in Western populations across Northern Europe and North America, whereas oesophageal squamous cell carcinoma (ESCC) show higher prevalence across Asian populations, particularly in China, India and Iran.^2^ Within China's epidemiological landscape, ESCC accounts for over four‐fifths of all EC diagnoses.^3^ Currently, surgical resection, radiotherapy, chemotherapy and immunotherapy remain the primary treatment strategies for ESCC. However, owing to its aggressive nature, high recurrence rate and early metastasis, the 5‐year survival rate for patients remains below 20%, resulting in a poor overall prognosis.^4^ The treatment of advanced ESCC relies mainly on guideline‐recommended systemic therapies, yet only a subset of patients experience significant benefit, while many experience limited efficacy with first‐line treatments.^2^

Despite significant advances in understanding cancer development, metastasis and drug resistance through in vitro models, translating these insights into clinical practice remains a major challenge. This limitation arises mainly from the inability of many existing cancer models to accurately replicate the complexity and heterogeneity of human tumours.^5^, ^6^, ^7^, ^8^ As a result, numerous compounds that demonstrate promising efficacy in preclinical cancer models often fail to achieve favourable outcomes in clinical trials.^9^ Organoids, three‐dimensional (3D) cell culture based in vitro models, offer an advanced system that closely mimics the structural and functional properties of native tissues or organs in vivo.^10^ They have been successfully established in various human cancers, including colon cancer,^11^ pancreatic cancer,^12^, ^13^ prostate cancer,^14^ liver cancer^15^ and breast cancer.^16^ By retaining the genetic, phenotypic and behavioural characteristics of their tissue of origin, tumour organoids have emerged as a highly promising platforms for drug discovery, treatment response evaluation and precision medicine research.^10^, ^17^, ^18^, ^19^, ^20^

Growing evidence underscores the considerable potential of tumour organoids in advancing personalised medicine. A 2018 study published in *Science* demonstrated that tumour organoid models could predict the clinical responses of patients with gastrointestinal cancer to chemotherapy. Organoid‐based drug sensitivity test (DST) exhibited high predictive accuracy, with a sensitivity of 100%, specificity of 93%, positive predictive value of 88% and negative predictive value of 100%.^21^ These findings suggest that tumour organoid models can be used to preemptively identify ineffective treatments, conserving valuable time and enabling the redirection of therapies toward options with a higher likelihood of efficacy. Subsequent studies have further reinforced the strong correlation between organoid‐based DST and patient clinical responses.^17^, ^18^, ^19^, ^22^, ^23^, ^24^ Encouragingly, Chen et al. demonstrated that high‐throughput drug screening with patient‐derived organoids (PDOs) produced significant therapeutic benefits when drugs identified as sensitive in vitro were translated into clinical use. Notably, this study confirmed that certain drugs initially approved for other malignancies (for example, bortezomib for multiple myeloma) exhibited significant anticancer effects in breast cancer organoid models. When these drugs applied to corresponding breast cancer patients, some advanced‐stage individuals experienced clinical downstaging and regained eligibility for surgical intervention.^25^

Despite growing interest in ESCC‐derived organoid models, reports remain limited.^22^ Kijima et al.^26^ generated organoids from 15 ESCC patients, but these were not applied to comprehensive genetic analysis or drug screening. Nakagawa^27^ subsequently established an ESCC‐derived organoid library from 24 patients and identified activation of the anti‐oxidative stress response as a key biomarker for chemoresistance. However, neither study addressed the urgent need to accelerate drug screening to align with the critical clinical decision‐making window. In response, this study proposed an innovative framework that uses ESCC‐derived tumour organoids as in vitro substitutes to establish an efficient two‐step drug screening method, aiming to evaluate multiple compounds within 30 days (the interval between surgery and the next treatment cycle). Analogous to clinical antibiotic sensitivity testing, this ‘trial drug’ approach enables clinicians to design precise, individualised treatment regimens for patients.

## 2 METHODS

### 2.1 Patient information

Clinical specimens of ESCC and matched non‐cancerous tissues were collected from individuals undergoing EC resection and reconstruction at the First Affiliated Hospital of Soochow University (August 2023–September 2024). Each diagnosis was confirmed by three independent pathologists using routine haematoxylin and eosin (H&E) and immunohistochemistry (IHC) staining. All participants provided signed informed consent in compliance with Helsinki Declaration ethical principles, with study protocols approved by the hospital's Ethics Committee (Approval No. 2023‐337). Inclusion requirements comprised: (1) Adult patients over 18 years; (2) tumour tissue diagnosed as ESCC and confirmed by routine H&E and IHC staining; (3) voluntary written informed consent; (4) locally advanced disease who meeting surgical indications following multidisciplinary consultation; (5) receipt of perioperative systemic chemotherapy. The exclusion criteria included the following: (1) other types of EC (for example, EAC) or non‐tumourous oesophageal diseases; (2) infectious diseases such as HIV, hepatitis B or syphilis; (3) early‐stage disease not requiring adjuvant therapy; (4) absence of perioperative adjuvant chemotherapy during follow‐up (for example, patients receiving only perioperative immunotherapy or radiotherapy). Detailed patient information is presented in Table S1.

Specimens were preserved in Primary Tissue Storage Solution (BioGenous, Jiangsu, China) within 30 min of collection and transported to the laboratory on ice. Tumour specimens were washed three times with ice‐cold Dulbecco's phosphate‐buffered saline (DPBS; Procell, China). Each tumour sample was then divided into multiple sections (.5–.8 cm) for histological analysis, ribonucleic acid (RNA) extraction, DNA extraction or dissociation for organoid culture.

### 2.2 Establishment and culture of ESCC organoids

Tumour tissues were dissected into minute fragments (approximately .5–1 mm in diameter) using sterile scissors, followed by triple DPBS washes at 4°C. The fragments were then digested in tumour tissue digestion solution (BioGenous) at 37°C for 30–60 min, until most cells were released into suspension. The digested material was dispersed into a suspension medium and passed through a 100 µm nylon cell strainer. This cellular mixture underwent centrifugation at 300×*g* for 5 min at 4°C, with subsequent DPBS washing cycles performed twice. For erythrocyte removal, the prepared cells were treated with 1× RBC lysis solution (Thermo Fisher Scientific) through gentle agitation for 10 min at 4°C, followed by additional DPBS rinses. The resulting cell suspension was mixed with organoid culture extracellular matrix (ECM; BioGenous) and plated into 24‐well plates. The plates were incubated at 37°C for 30–40 min to allow the ECM to solidify. After solidification, 500 µL of organoid complete medium (EC Organoid Kit; BioGenous) was carefully added to each well. The culture medium was refreshed twice weekly to support optimal organoid growth and maintenance. The growth and morphology of the organoids were monitored using an inverted microscope. Depending on growth status, organoid cultures were passaged at a 1:1.5–3 dilution every 7–14 days after incubation in organoid dissociation solution (BioGenous) for 15–40 min.

### 2.3 Lentiviral transduction and stable cell line generation

Plasmids for constructing stable cell lines, including OE BATF2, EV BATF2, sh BATF2, NC BATF2, OE INPPL1, EV INPPL1, sh INPPL1 and NC INPPL1, were purchased from Miaolingbio (Wuhan, China). Lentivirus was prepared by transfecting the corresponding plasmids according to a previously reported protocol.^28^ Organoids were dissociated into single cells, and lentiviral transduction was performed using a low‐speed centrifugation method (1000–1200×*g* for 1 h). Afterward, cells were maintained in viral supernatant‐containing medium with fresh viral supernatant added the following day. Puromycin selection commenced 72 h post‐transduction, with resistant organoids subsequently isolated and confirmed through western blot analysis using antibodies detailed in Table S2.

### 2.4 Clinical response assessment

All participants underwent perioperative adjuvant chemotherapy and were divided into two groups based on whether they had received neoadjuvant chemotherapy: The neoadjuvant therapy (NAT) group and the treatment‐naïve group (those who did not receive preoperative chemotherapy). Clinical responses to neoadjuvant or adjuvant therapy were evaluated using imaging and pathological evaluations.

In the NAT group, histological response to chemotherapy was evaluated according to the criteria of the Japanese Society for Esophageal Disease (JSED),^29^ as outlined in Table 1. Pathological evaluation of treatment efficacy was performed in a blinded manner by three independent gastrointestinal pathologists. A histological response of Grade 2 or 3 in the primary tumour was classified as a good response, whereas a response of Grade 0 or 1 was categorised as a poor response.

**TABLE 1:** Histological response criteria for chemotherapy and/or radiotherapy established by the Japanese Society for Esophageal Disease.

Grade	Description
Grade 0: Ineffective	No recognisable cytological or histological therapeutic effect
Grade 1: Slightly effective Grade 1a: Viable cancer cells account for 2/3 or more tumour area Grade 1b: Viable cancer cells account for ≥1/3 but <2/3 of the tumour area	Viable cancer cells (including those with eosinophilic cytoplasm with vacuolation and swollen nuclei) account for 1/3 or more of the tumour tissue; however, there is some evidence of degeneration of cancer tissue or cells.
Grade 2: Moderately effective	Viable cancer cells account for <1/3 of the tumour area, whereas other cancer cells are severely degenerated or necrotic.
Grade 3: Markedly effective	No viable cancer cells are evident.

In the treatment‐naïve group, clinical responses to postoperative adjuvant chemotherapy were assessed during follow‐up. Follow‐up evaluations included regular imaging examinations (computed tomography [CT] or positron emission tomography), endoscopic evaluations and serum tumour marker testing. A good clinical response was defined as the absence of residual tumour or lymphadenopathy on imaging or endoscopy, along with tumour marker levels within normal ranges. Conversely, if abnormalities were detected in imaging or endoscopic evaluations, or if tumour marker levels were elevated, the clinical response was classified as poor. Follow‐up visits were typically scheduled every 3–6 months during the first 2 years after surgery and annually thereafter with adjustments made according to the patient's condition.

### 2.5 Histological assessment

Tissues and organoids were preserved in 4% paraformaldehyde before being processed into 4 µm‐thick sections. H&E staining was performed according to standard histological protocol. Immunohistochemical staining was conducted using the following antibodies: Anti‐Ki67 (1:400; Biolynx; BX50040), anti‐p53 (1:200; Proteintech; 10442‐1‐ap), anti‐p40 (1:200; Biolynx; BX50028) and anti‐p63 (1:200; Biolynx; BX50033). Apoptosis was assessed through terminal deoxynucleotidyl transferase nick‐end labelling (TUNEL) staining using the KeyGen BioTECH KGA700 detection kit. For quantification, five random fields per specimen were imaged at 40× magnification, and the percentage of positively stained cells was quantified using ImageJ software.

### 2.6 Whole exome sequencing and data analysis

Whole exome sequencing (WES) was conducted on DNA extracted from tumour organoids, tumour tissue samples and adjacent non‐tumourous oesophageal mucosa from four patients (Patient IDs: ESC1, ESC22, ESC23 and ESC24) for WES. Sample selection was based on tumour size to ensure sufficient material for sequencing, organoid culture and pathological analysis, while preserving tissue required for postoperative diagnoses. Larger tumour samples were prioritised to meet these requirements. Genomic DNA isolation employed Qiagen's DNeasy & RNeasy Isolation Kit, followed by electrophoretic quality verification prior to sequencing preparation. Genomic DNA underwent mechanical shearing via a Covaris system to generate fragments ranging from 150 to 220 base pairs. Library preparation and target enrichment were carried out using the Agilent SureSelect Human All Exon V8 capture system. High‐throughput sequencing was conducted using the Illumina NovaSeq 6000 system, yielding 150‐bp paired‐end sequencing reads. Raw sequencing data underwent quality control to obtain clean reads, with preprocessing performed using FastQC. Clean reads were aligned to the reference genome using Burrows‐Wheeler Aligner. Alignment results were converted into the appropriate format using SAMtools, deduplicated using Picard (http://broadinstitute.github.io/picard) and assessed with Qualimap software. The reference genome used was GRCh37.p13, available at: https://ftp.ncbi.nlm.nih.gov/genomes/all/GCF/000/001/405/GCF_000001405.25_GRCh37.p13/GCF_000001405.25_GRCh37.p13_genomic.fna.gz.

Single‐nucleotide polymorphisms and insertions or deletions (InDels) were detected from the sample alignments to the reference genome using the HaplotypeCaller module of GATK4. Variants annotation was performed with databases including Refseq, 1000 Genomes, EXAC, esp6500, gnomAD, SIFT, ClinVar, PolyPhen, MutationTaster, COSMIC, GWAS Catalog and OMIM using Annova. Copy number variations (CNVs) were identified with Control‐FREEC, comparing normal tissue and paired tumour or organoid samples to detect somatic CNVs.

### 2.7 RNA sequencing and data analysis

Nucleic acid extraction utilised Qiagen's DNeasy & RNeasy Isolation Kit followed by poly‐T bead‐based mRNA enrichment. RNA libraries were prepared with the VAHTS Universal V8 RNA‐Seq Library Prep Kit, and sequenced on the Illumina HiSeq X Ten platform. Gene expression levels were quantified using featureCounts or StringTie and normalised to fragments per kilobase of transcript per million mapped reads or transcripts per million. Differential expression analysis between drug‐resistant and drug‐sensitive groups was performed using DESeq2 or edgeR, with significance defined as false discovery rate < .05 and a log_2_ fold change ≥ 1. Differentially expressed genes (DEGs) were annotated with the Kyoto Encyclopedia of Genes And Genomes (KEGG) database to identify associated biological pathways. Enrichment of pathways was conducted using the clusterProfiler package with hypergeometric testing, applying a significance threshold of *p* < .05. Results were visualised using bubble plots or bar charts to display pathway significance, gene counts and enrichment fold change. Co‐expression network construction and module detection were implemented via the WGCNA package in R, enabling identification of biologically meaningful correlated gene clusters.^30^

### 2.8 Drug screening procedure

In this study, 10 anti‐tumour compounds were used for drug screening (Table 2). Each compound was dissolved in either H_2_O or DMSO according to the manufacturer's instructions and stored at −80°C. The preliminary screening concentrations were set to the maximum plasma concentration (*C*_max_) for each drug. *C*_max_ values were obtained by retrieving relevant drug information from DrugBank (https://go.drugbank.com/), and referencing the *C*_max_ values reported in the associated literature.^31^, ^32^, ^33^, ^34^, ^35^, ^36^, ^37^, ^38^, ^39^, ^40^ For the secondary screening, each drug was tested in triplicate across a six‐point, fourfold serial dilution, with different starting concentrations (Table 2). The concentration used in the secondary screening (for example, doxorubicin 50 µM) was based on previously published literature.^41^

**TABLE 2:** The 10 anti‐tumour compounds used in drug screening and their preliminary screening concentrations, along with the starting concentrations for secondary screening.

Drug	Preliminary screening concentration (µmol/L)	Secondary screening concentration (µmol/L)
Cisplatin	13.67	50
Paclitaxel	5.10	50
Fluorouracil	7.5	50
SN‐38	.15	5
Fedratinib	3.44	50
Gemcitabine	101.04	750
Epirubicin hydrochloride	.16	5
Doxorubicin hydrochloride	6.90	50
Palbociclib	.22	5
Docetaxel	2.98	50

Tumour organoids were digested with organoid passaging digestion solution at 37°C for 15–20 min, until most organoids exhibited dissociation into single cells, as observed microscopically. The cells were then washed twice with cold DPBS (4°C), filtered through a 70 µm nylon cell strainer, centrifuged and resuspended in ECM.

For the two‐step drug screening procedure, organoid suspensions were plated in 96‐well plates (for preliminary screening) and 24‐well plates for further expansion culture during secondary screening. After 20 min of solidification, 100 µL of culture medium was added to the 96‐well plate. Approximately 2 days later, when organoid cells had reformed spherical structures, the medium was replaced with drug‐containing culture medium. In the experiment, .1% DMSO served as the negative control (NC), and 200 µL of sterile water was added to the surrounding wells to minimise evaporation. After approximately 4 days of drug treatment, cell adenosine triphosphate (ATP) levels were measured using the CellTiter‐Glo 3D assay (G9683; Promega) according to the manufacturer's instructions. In the preliminary screening, organoid viability was calculated as a ratio of drug‐treated group to the NC group. Candidate drugs for secondary screening were identified based on the following selection criteria: Cell viability ≤ 20% and selection of the three agents with the most significant inhibitory effects. During the secondary screening phase, organoids seeded in 24‐well plates were expanded for 4 days and then reseeded into 96‐well plates using the same method as the preliminary screening. After identifying candidate drugs, the culture medium was replaced with fresh medium containing these drugs (the three with the strongest inhibitory effects and the taxol + cisplatin [TP] regimen). ATP values were measured at the start and end of treatment to calculate the growth rate inhibition (GR) value.

In traditional drug screening, tumour organoids require expansion and long‐term culture to generate sufficient cell quantities for multi‐drug testing, a process similar to that used in secondary screening. The expanded organoid suspension was plated into 96‐well plates, followed by medium addition and pre‐culture. Organoids were then treated with media containing 10 drugs, each tested at six concentrations in a fourfold dilution series and in triplicate. After treatment, ATP levels were measured using the CellTiter‐Glo 3D assay, and the inhibitory effects on cell viability were calculated. Dose–response curves were analysed to determine GR values for all drugs.

GR values were calculated using R package GR metrics.^42^ Data were analysed using three‐parameter logarithmic regression fitting in GraphPad Prism (version 9.0) software, with slope variation set and the activity range constrained between 100 and 0%. The area under the curve (AUC) was computed from the raw data and standardised by dividing the maximal possible AUC within the 0–100% activity range. The final AUCsum was calculated as the sum of the standardised AUCs for cisplatin and paclitaxel (PTX). All reagents and their sources are listed in Table S2.

### 2.9 Caspase‐3/7 activity assay

Organoids were treated with complete culture medium containing cisplatin (1 µM), PTX (1 µM) or .1% DMSO for 48 and 96 h. After the treatment periods, the original culture medium was aspirated, and 5 µM CellEvent™ caspase‐3/7 green detection reagent (Thermo Fisher Scientific, USA) was added to the organoid cultures. Organoids were incubated in a complete culture medium at 37°C for 30 min. Staining was observed using fluorescence microscopy, with five random areas captured at 10× magnification. The percentage of positively stained areas was quantified using ImageJ software.

### 2.10 Patient‐derived tumour organoid xenograft model and chemotherapy in BALB/c nude mice

This study employed 3–5 week‐old female BALB/c nude mice from Soochow University's Experimental Animal Center for developing subcutaneous tumour models. All experiments were approved and monitored by the same centre (Animal Welfare Assurance no. 202408A0242), in accordance with animal welfare guidelines and the ‘3Rs’ principle. Two drug‐resistant organoid lines (ESC22‐PDO and ESC30‐PDO) were cultured to sufficient cell numbers and counted. Cells were resuspended in DPBS and mixed thoroughly with an equal amount of matrix gel. The cell suspension was aspirated into a 1 mL sterile syringe and subcutaneously injected into the left posterior flank of 5‐week‐old nude mice (3–4 million cells per mouse). Tumour volume was measured every 5 days using a digital caliper and calculated as volume = length × width2 × .5. When the tumour volume reached 50 mm^3^, the mice were randomly assigned to one of three treatment groups: NC, TP regimen or doxorubicin. Drugs were administered through intraperitoneal injection as follows: The NC group (.9% NaCl, twice weekly), TP regimen (cisplatin 5 mg/kg + PTX 10 mg/kg, dissolved in .9% NaCl, twice weekly) and the doxorubicin group (2 mg/kg, dissolved in .9% NaCl, twice weekly). Tumour diameter was maintained below 150 mm. After euthanasia, tumours were excised, photographed, weighed and were fixed in 4% paraformaldehyde for IHC and immunofluorescence analyses.

### 2.11 Cell lines and culture conditions

The human lung adenocarcinoma cell line A549 (FH0045) and its drug‐resistant derivatives A549/DDP (cisplatin‐resistant, FH0091) and A549/Tax (PTX‐resistant, FH0609) were obtained from Fuheng Biotechnology Co., Ltd (Shanghai, China). Cells were maintained in RPMI‐1640 medium (Gibco) supplemented with 10% foetal bovine serum (Gibco) and 1% penicillin–streptomycin at 37°C in a humidified incubator with 5% CO_2_.

### 2.12 Quantification and statistical analysis

Statistical analyses were conducted using GraphPad Software Inc. (San Diego, CA) and R programming environment. For comparisons between two independent groups, two‐tailed unpaired Student's *t*‐tests were applied, while experimental groups numbering three or more were analysed through one‐way ANOVA. Quantitative data are expressed as mean ± standard deviation, with statistical significance determined when probability values fell below the .05 threshold.

## 3 RESULTS

### 3.1 Establishment and validation of a living ESCC organoid biobank

**FIGURE 1:**
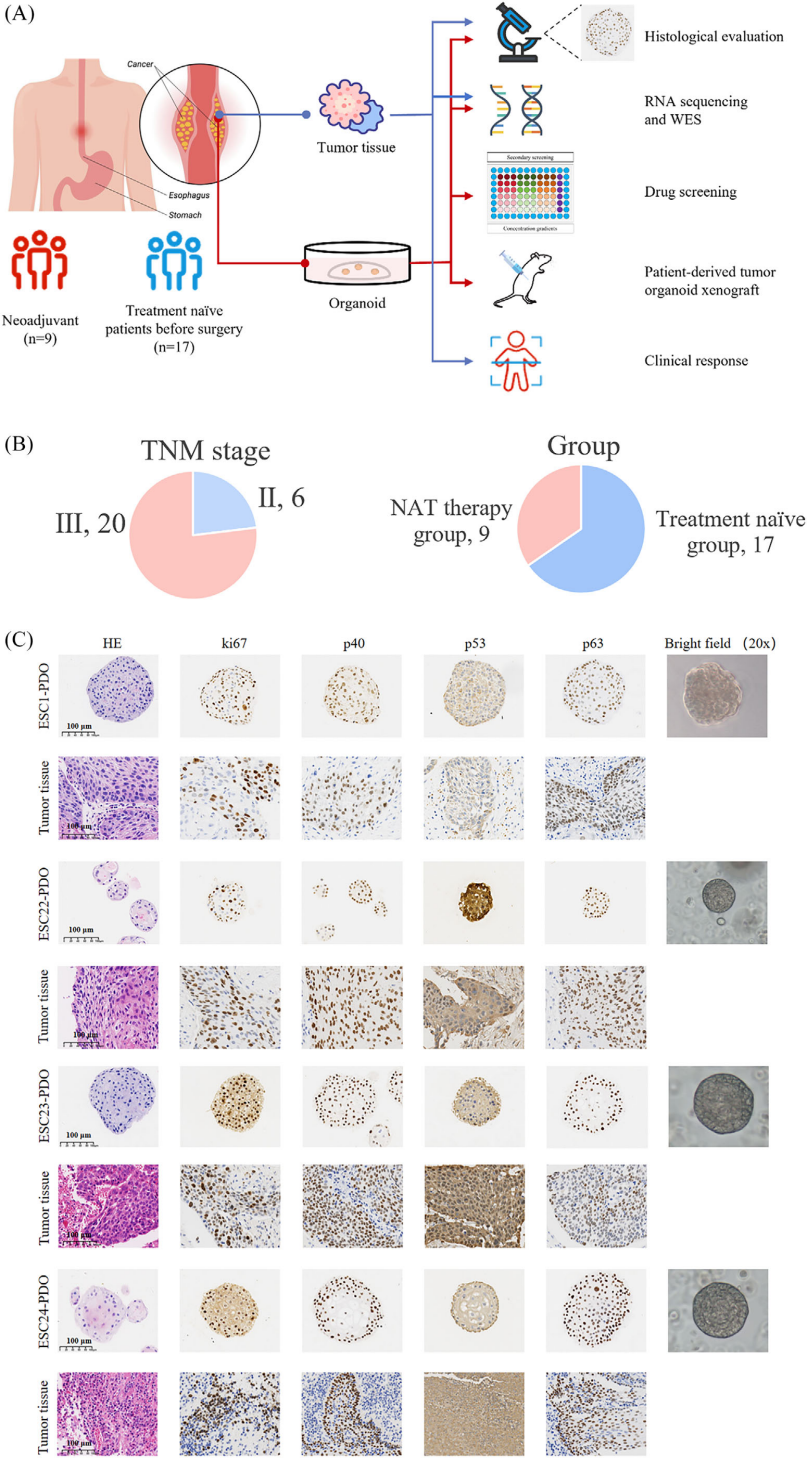
Experimental design and histological analysis of organoids. (A) Schematic overview of the study design. Fresh ESCC samples were obtained from patients (Table S1) and processed as described in *Methods* section. (B) Pie charts illustrating the distribution of patients by tumour–node–metastasis stage (left) and by group assignment (right). (C) Histological and immunohistochemical analysis of ESCC organoids and corresponding tumour tissues. The figure shows H&E staining, IHC staining and 20× magnification images for four pairs of ESCC organoids (from top to bottom: ESC1‐PDO, ESC22‐PDO, ESC23‐PDO, ESC24‐PDO) and their corresponding tumour tissues. Scale bars, 100 µm. *Abbreviation*: PDO, patient‐derived organoid.

From August 2023 to September 2024, we successfully established 26 PDO cultures from locally advanced ESCC patients undergoing perioperative chemotherapy. Patients were divided into two groups based on whether they received neoadjuvant chemotherapy before surgery: the NAT group (*n* = 9) and the treatment‐naïve group (*n* = 17) (Figure 1A). These PDOs were derived from surgical resection specimens of patients, with stage II (*n* = 6) and stage III (*n* = 20) ESCC (Figure 1B). Detailed clinical information for these patients is provided in Table S1. In the NAT group, organoid cultures were successfully established from 9 to 13 ESCC samples, yielding a success rate of 69.2%. The primary reasons for failure included: Three patients with postoperative pathological staging of ypT0N0M0, indicating complete tumour regression following NAT (accounting for 75% of failures), and bacterial contamination in one case (accounting for 25%). In the treatment‐naïve group, organoid cultures were successfully established in 17 out of 25 cases, with a success rate of 68%. The main reasons for failure were insufficient viable tumour cells to sustain long‐term passaging (four cases, 50% of failures) and bacterial contamination (four cases, 50% of failures).

We selected four pairs of organoids (ESC1, ESC22, ESC23 and ESC24) and their corresponding parental tumour tissues for H&E and IHC staining, followed by paired comparison (Figure 1C). H&E staining revealed that the organoids closely resembled the parental tumour tissue in terms of cellular morphology and structural characteristics, displaying typical histological features of ESCC, including tightly packed cells, abundant mitotic figures and irregularly shaped tumour cells. In subsequent IHC staining, we assessed four key ESCC‐related markers: Ki67, p40, p53 and p63. Ki67, a well‐established marker of cell proliferation,^43^ and p40 and p63, typical nuclear markers for ESCC, are commonly used in the pathological diagnosis.^44^ p53, a tumour suppressor gene, is frequently aberrantly expressed or mutated in tumour tissues. The expression patterns of Ki67, p40, p53 and p63 in the organoids matched those in the corresponding tissues (Figure 1C).

**FIGURE 2:**
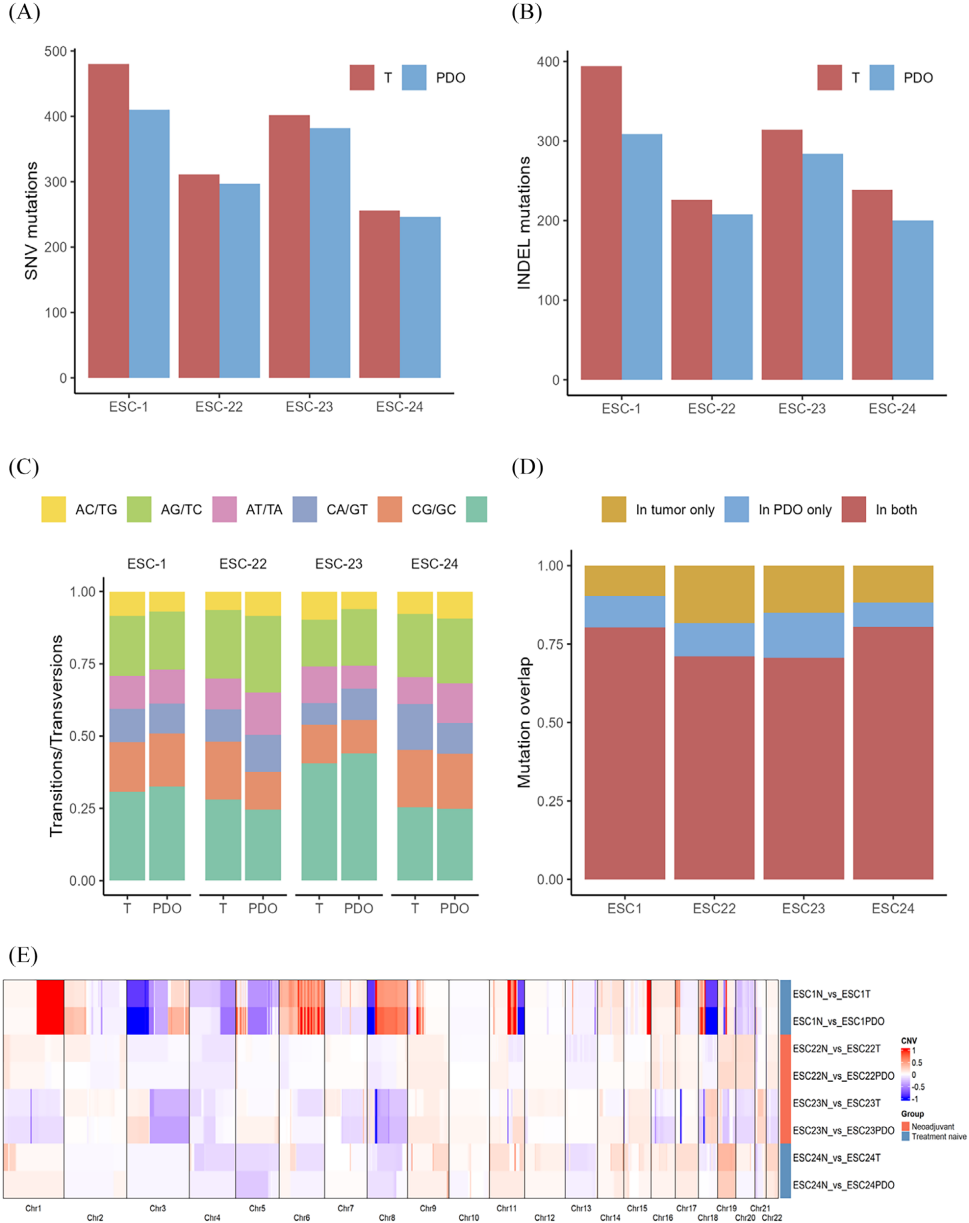
Genomic mutation analysis of PDOs and matched tumour tissues. (A) Number of single nucleotide variations (SNVs) in four samples (ESC1, ESC22, ESC23 and ESC24), with red bars representing tumour tissue and blue bars representing organoids. (B) Number of insertion/deletion (InDels) mutations in tumour tissue versus organoids. (C) Distribution of nucleotide transitions and transversions. (D) Overlap of SNV mutations between organoids and tumour tissues, with red indicating shared mutations by both, yellow representing tumour‐specific mutations and blue representing organoid‐specific mutations. (E) Copy number variation (CNV) profiling of organoids, tumour tissue and normal tissue. *Abbreviations*: PDO, patient‐derived organoid; T, tumour tissue; N, normal tissue; CNV, copy number variation.

To assess whether the PDO model retains the genetic characteristics of the parental tumours at the genomic level, we performed WES on four selected organoids (ESC1‐PDO, ESC22‐PDO, ESC23‐PDO and ESC24‐PDO), along with their matched tumour and normal tissues. Samples for WES or RNA seq were selected based on their tumour size and material availability. Only those with sufficient DNA or RNA quantity and quality, ensuring compatibility with both sequencing and organoid culture, were selected. Additionally, care was taken to ensure that sample collection did not interfere with postoperative pathological diagnoses. Analysis of single nucleotide variations (SNVs) (Figure 2A) and InDels (Figure 2B) revealed that, although there were some differences in the total number of mutations between the organoids and the primary tumour tissues, the majority of mutations were preserved. Moreover, the ratio of transitions and transversions indicated a high similarity in the mutation type distribution between the two (Figure 2C). Overlap analysis demonstrated that the median overlap of SNV mutations was 75.7% (range: 70.5–80.6%) (Figure 2D). Additionally, the CNV heatmap displayed that the PDOs closely resembled the tumour tissues in the overall distribution of genomic structural variations, preserving the major CNV features (Figure 2E). Mutation analysis of key driver genes revealed different types of mutations, including frameshift, nonsense, missense and in‐frame insertions or deletions. The PDOs and their parental tumours exhibited a high degree of similarity in mutation types and distribution for key driver genes, such as TP53, KMT2D, NOTCH1 and MUC16 (Figure S1A). The observed differences in mutation patterns between samples reflected the inter‐tumoural heterogeneity (Figure S1A). To assess transcriptional similarity, we performed RNA sequencing (RNA‐Seq) on 11 paired PDOs and matched primary tumours. The correlation matrix revealed a high RNA expression correlation, with a median coefficient of 87.21% (range: 68.81–96.65%; Figure S1B and Table S3).

Taken together, ESCC organoids established in this study successfully recapitulated histological, genomic and transcriptomic features of their parental tumour tissues.

### 3.2 Two‐step drug screening using GR values accurately reflects clinical response

We developed a streamlined two‐step drug screening method to generate reliable results for 10 compounds within 30 days. The procedure is illustrated in Figure 5A, with detailed steps described in *Methods* section. In the secondary screening, GR metrics were used instead of traditional metrics such as IC_50_ to evaluate organoid responses to drugs across concentration gradients. This choice was based on studies revealing that traditional metrics such as IC_50_ and *E*_max_ often lack consistency across experiments and are unreliable for predicting drug sensitivity.^45^, ^46^, ^47^ Hafner et al.^42^ identified inherent flaws in traditional methods, which tend to overestimate drug tolerance when cell division is limited during assays. These errors arise from the reliance on cell count measurements, which can be influenced by experimental variables such as seeding density, medium composition and cell division rate. The GR metric, ranging from −1 to 1, normalises growth rates to account for division rate variability, providing a more accurate and reliable assessment of drug response.^48^

In this study, all patients were followed from surgery until the last clinical evaluation or dropout, with a median follow‐up of 16 months (range: 13–18 months). Chemotherapy efficacy was assessed in both the NAT and treatment‐naïve groups, as described in *Methods* section. Follow‐up data were missing for two patients (ESC11 and ESC28). Detailed treatment regimens and clinical response information are provided in Table S1. Figure S2 presents cell viability results for 26 ESCC organoids at the preliminary screening concentration. The *C*_max_ of the drugs is detailed in Table 2. Comprehensive cell viability data for the different organoids under various drug treatments are provided in Table S4. Overall, preliminary screening results indicate notable variability in drug responses across organoids from different patients.

**FIGURE 3:**
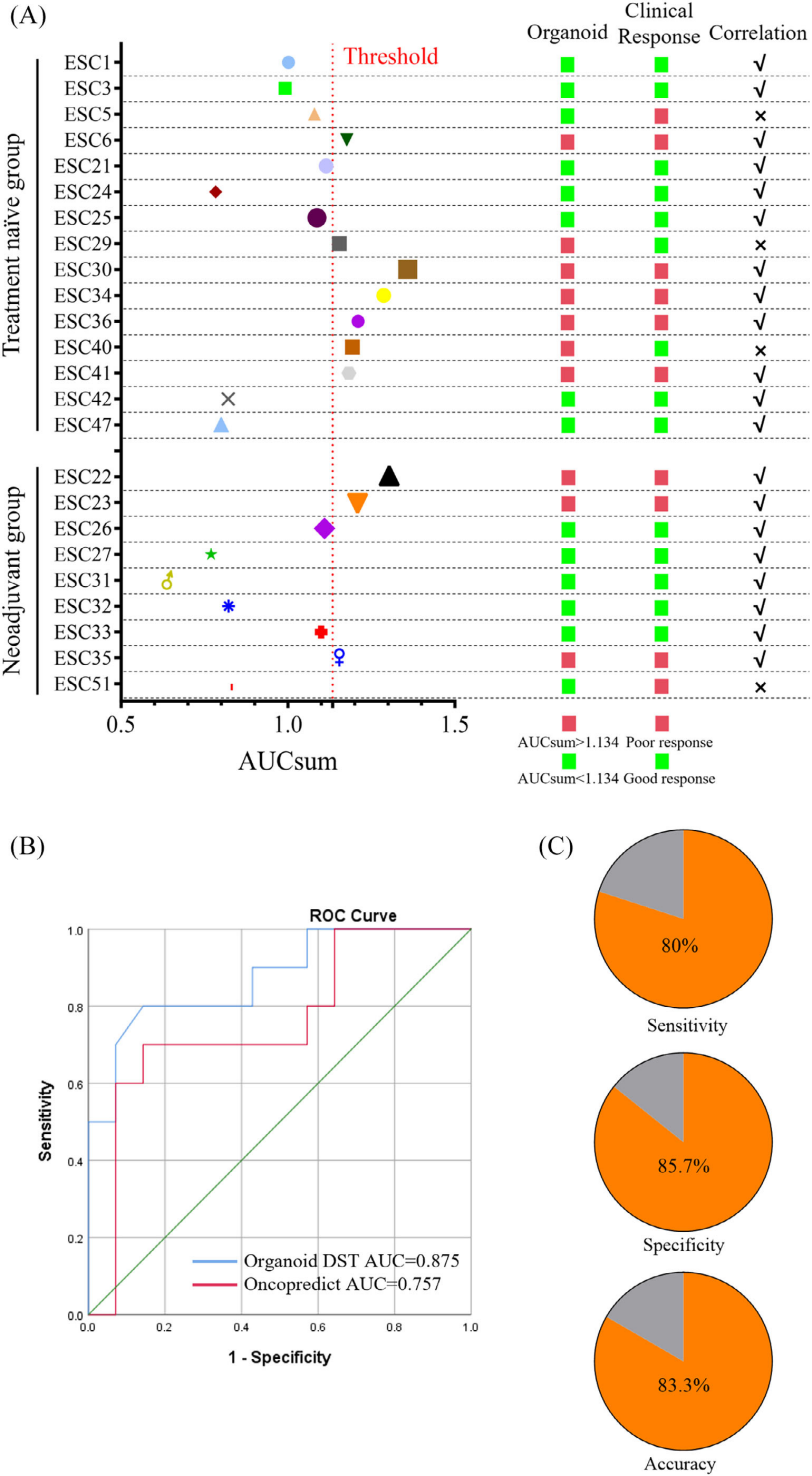
Consistency between organoid‐based DST and clinical treatment response. (A) Correlation analysis between organoid‐based DST and clinical treatment response. AUCsum represents the combined of cisplatin and PTX in the organoid DST, with the threshold (red dashed line) set at 1.134 √ indicates organoid DST consistent with clinical treatment outcome, whereas × indicates inconsistency. (B) ROC curve depicting the performance of organoid DST and transcriptomic prediction using OncoPredict. (C) Pie charts summarising organoid DST performance.

Based on the preliminary screening results, drugs displaying the most effective inhibition (selection criteria: cell viability ≤20%, and the top three most inhibitory drugs) were selected for secondary screening alongside common perioperative TP chemotherapy agents (cisplatin and PTX). The results are presented in Table S5. Figures S3A and S4A display the dose–response curves for cisplatin and PTX, respectively, across organoids derived from different patients. The data reveal significant variability in drug sensitivity among organoids from different patients. Strong correlations between the standardised AUC values (cisplatin: Figure S3B; PTX: Figure S4B) and GR metrics (cisplatin: Figure S3C; PTX: Figure S4C) indicate the robustness of both measures in assessing drug response (cisplatin: *R* = .989, *p *< .0001; Figure S3D; PTX: *R* = .995, *p* < .0001; Figure S4D). We then calculated the standardised AUC values for cisplatin and PTX as detailed in *Methods* section. Because these two drugs are commonly used in combination in clinical practice, their standardised AUC values were summed to obtain AUCsum. Figure 3A illustrates the correlation analysis between organoid DST and clinical response in the chemotherapy‐naïve and NAT groups. We then applied the OncoPredict algorithm^49^ to estimate organoid responses to cisplatin and PTX based on transcriptomic profiles. The predicted drug sensitivities were compared with clinical outcomes using a binary logistic regression model. As shown in Figure 3B, the OncoPredict‐based model achieved an area under the ROC curve (AUC) of .757. In contrast, when drug responses were assessed using the AUCsum threshold of 1.134, the ROC analysis yielded a higher AUC of .875, demonstrating superior discriminative performance. These findings highlight the superior predictive power of PDO‐based DST compared with traditional tumour biomarker‐based approaches. Pie charts further summarise the statistical metrics at this cutoff: Sensitivity, 80%; specificity, 85.7%; and overall accuracy, 83.3% (Figure 3C). To examine whether heterogeneous treatment histories influence the drug response profiles of PDOs, we performed principal component analysis using transcriptomic data. As shown in Figure S5A, PDOs from NAT and treatment‐naïve patients did not form distinct clusters, indicating comparable global transcriptional landscapes between the two groups. In contrast, Figure S5B reveals a clear separation between PDOs with different AUCsum, supporting the robustness of this threshold in distinguishing drug‐resistant from drug‐sensitive organoids. Furthermore, as shown in Figure S5C, although a slightly higher proportion of treatment‐naïve PDOs exhibited enhanced drug sensitivity, the difference was not statistically significant (Fisher's exact test, *p* > .05). Together, these results suggest that diverse treatment backgrounds exert minimal influence on the overall transcriptomic and chemosensitivity landscapes of PDOs in this study. In contrast, PDOs stratified by AUCsum values displayed distinct clustering in transcriptomic analyses, emphasising the strong discriminatory power of AUCsum in capturing intrinsic drug response differences.

**FIGURE 4:**
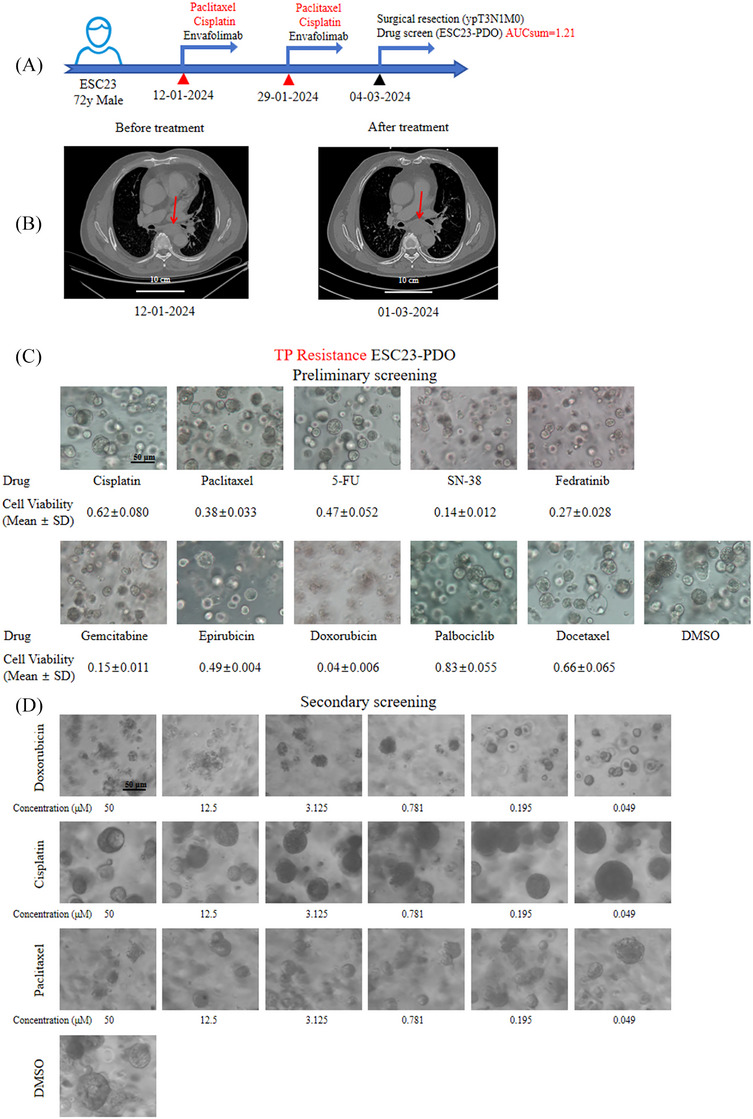
Treatment course, imaging evaluation before and after NAT and organoid drug sensitivity screening experiment of the ESC23 patient. (A) Treatment timeline of the ESC23 patient. (B) CT images of the ESC23 patient before and after NAT. The tumour location is indicated by the red arrow. Scale bars, 10 cm. (C) Preliminary drug screening results of ESC23‐PDO. Scale bars, 50 µm. (D) Secondary drug sensitivity screening of ESC23‐PDO, indicating cisplatin, PTX and doxorubicin (drugs with the highest *C*_max_/GR100 values). Scale bars, 50 µm.

In addition, organoid DST closely correlated with pre‐ and post‐treatment imaging findings in patients with NAT. ESC23, a 72‐year‐old male with ESCC, underwent two cycles of cisplatin combined with PTX and envafolimab as NAT before surgery in March 2024 (Figure 4A). CT imaging (Figure 4B) revealed an increase in tumour following NAT, with post‐surgical JSED pathological evaluation indicating Grade 1b, suggesting a limited therapeutic effect. Organoid DST revealed an AUCsum of 1.21, indicating resistance to cisplatin and PTX in vitro. Preliminary screening revealed weak efficacy for cisplatin, with cell viability of .62 ± .08, and PTX, with a cell viability of .38 ± .033 (Figure 4C). Secondary screening of the ESC23‐PDO (Figure 4D) revealed that doxorubicin's inhibitory effect diminished with decreasing concentration but retained some activity at low doses. Cisplatin and PTX demonstrated partial inhibition at higher concentrations, but their effects were limited, consistent with the clinical response. ESC27, a 66‐year‐old male with ESCC, received three cycles of cisplatin and PTX as NAT before surgery in February 2024 (Figure S6A). CT imaging (Figure S6B) indicated tumour shrinkage after therapy, and post‐surgical JSED pathological evaluation revealed Grade 2, suggesting a good therapeutic effect. Organoid DST revealed an AUCsum of .77, indicating relatively sensitivity to cisplatin and PTX in vitro. Preliminary screening revealed that cisplatin (cell viability .33 ± .036), exhibited a limited inhibitory effect, whereas PTX (cell viability .06 ± .005) demonstrated a strong inhibitory effect (Figure S6C). Secondary screening of the ESC27 organoid model (Figure S6D) evaluated the growth‐inhibitory effects of varying concentrations of doxorubicin, cisplatin and PTX. Doxorubicin caused significant organoid disintegration, indicating potent cytotoxicity. Cisplatin and PTX exhibited partial inhibition even at lower concentrations, as evidenced by organoid shrinkage and partial dissociation, aligning with the patient's clinical response. Figure S7 illustrates apoptosis levels and time‐dependent changes in drug‐sensitive (ESC27‐PDO) and drug‐resistant (ESC23‐PDO) organoids treated with cisplatin (1 µM) and PTX (1 µM), as assessed by Caspase 3/7 staining. Microscope images in Figure S7A illustrate weak apoptotic signals in the resistant ESC23‐PDO organoids, with only faint green fluorescence, indicating resistance to both cisplatin and PTX. In contrast, ESC27‐PDO organoids demonstrated strong green fluorescence following drug treatment, with fluorescence intensity increasing from day 2 to day 4, reflecting significant drug‐induced apoptosis. Quantitative analysis in Figure S7B further confirmed that apoptosis signals were significantly higher in ESC27‐PDO organoids compared with ESC23‐PDO.

### 3.3 Two‐step drug screening method significantly reduces DST duration to 1 month

**FIGURE 5:**
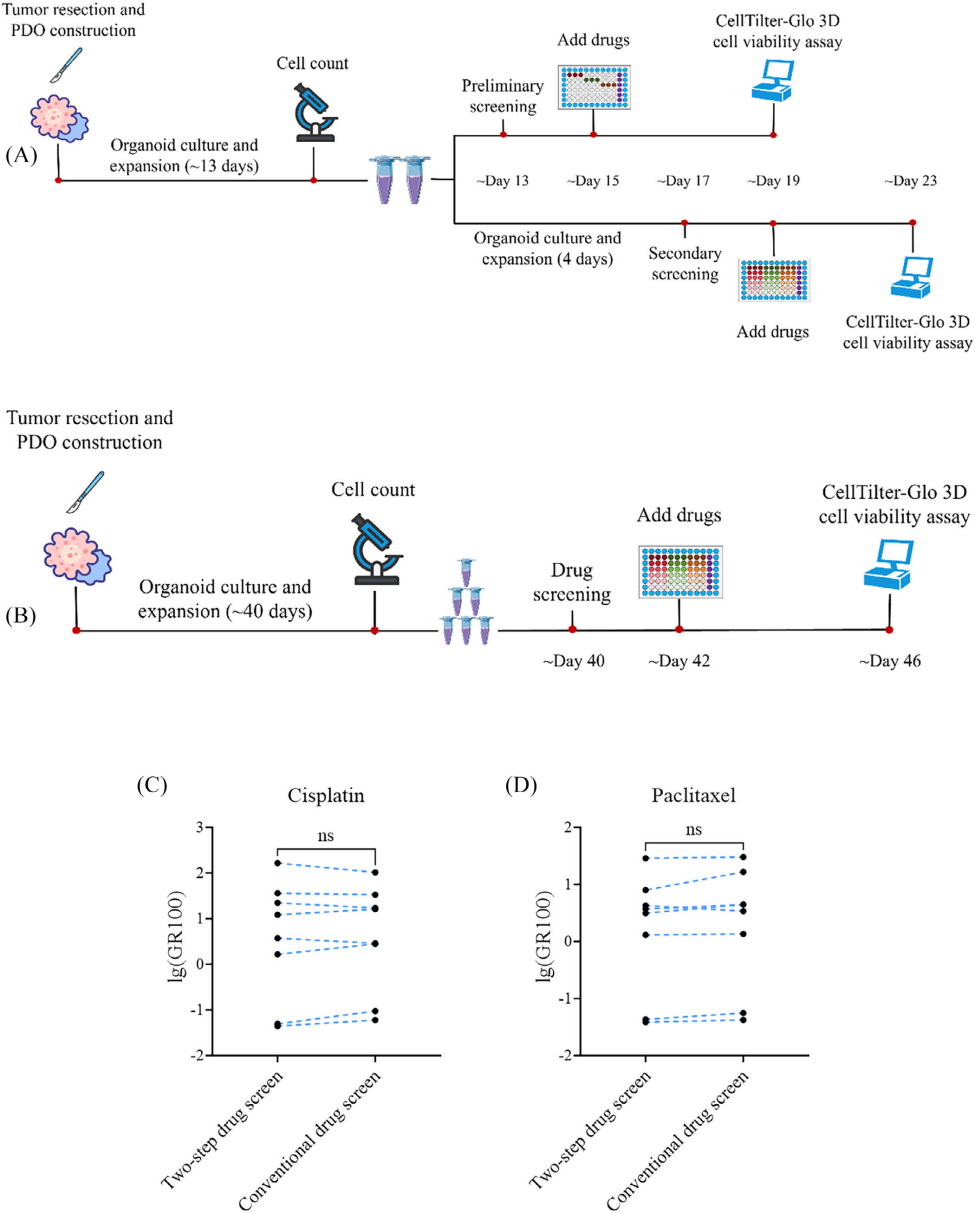
Comparison of the two‐step and the conventional drug screening methods. (A) Schematic of the two‐step drug screening process using organoid models. Detailed procedures are described in *Methods* section. (B) Schematic of the conventional drug screening process. Detailed procedures were described in *Methods* section. (C) Log‐transformed drug response (lg(GR100)) for cisplatin using the two‐step and conventional methods. (D) Log‐transformed drug response (lg(GR100)) for PTX using the two‐step and conventional methods.

To assess the advantages and clinical applicability of the two‐step drug screening method, we compared it with the traditional approach. Tumour tissues from eight patients (ESC6, ESC23, ESC24, ESC2, ESC28, ESC35, ESC40 and ESC41) were subjected to both screening methods simultaneously. The traditional method used six concentrations with fourfold dilutions, and triplicate experiments for all drugs. Details are indicated in Figure 5B with a full description provided in *Methods* section. As revealed in Table S6, experimental results were expressed by GR100 and the ratio of *C*_max_/GR100. GR100 ratio was chosen because it can be directly obtained from most dose–response curves, offering greater robustness and reliability than other GR metrics such as GR50, which require curve fitting. Comparison of the two methods for cisplatin (Figure 5C) and PTX (Figure 5D) revealed no significant difference in the lg(GR100) values, demonstrating the robust and reproducible drug sensitivity results of the two‐step screening method. Compared with the traditional method, the two‐step approach exhibited the following advantages (Table 3): lower cell requirements, fewer passages, reduced reagent consumption and a shorter time to results. Overall, the two‐step drug screening method not only replicates the drug response outcomes of traditional methods with reliability and reproducibility but also improves clinical applicability by conserving resources and reducing experimental time.

**TABLE 3:** Comparison of the two‐step drug screening method with the traditional drug screening method.

	Two‐step drug screen (*n* = 26)	Conventional drug screen (*n* = 8)	*p* Value
Number of passage			<.001
2	12 (46.2%)		
3	14 (53.8%)		
4		3 (37.5%)	
5		3 (37.5%)	
6		2 (25%)	
Number of cells (cells)	22.93 ± 7.73 × 10^4^	188.45 ± 50.77 × 10^4^	<.001
Duration from tissue receipt to drug screen result (days)	23.08 ± 2.42	45.75 ± 7.19	<.001
Consumption of matrigel (mL)	1.27 ± .05	3.40 ± .21	<.001
Consumption of total medium (mL)	46.18 ± 3.16	121.38 ± 7.33	<.001

### 3.4 Transcriptional signatures of ESCC PDOs associated with PTX resistance

**FIGURE 6:**
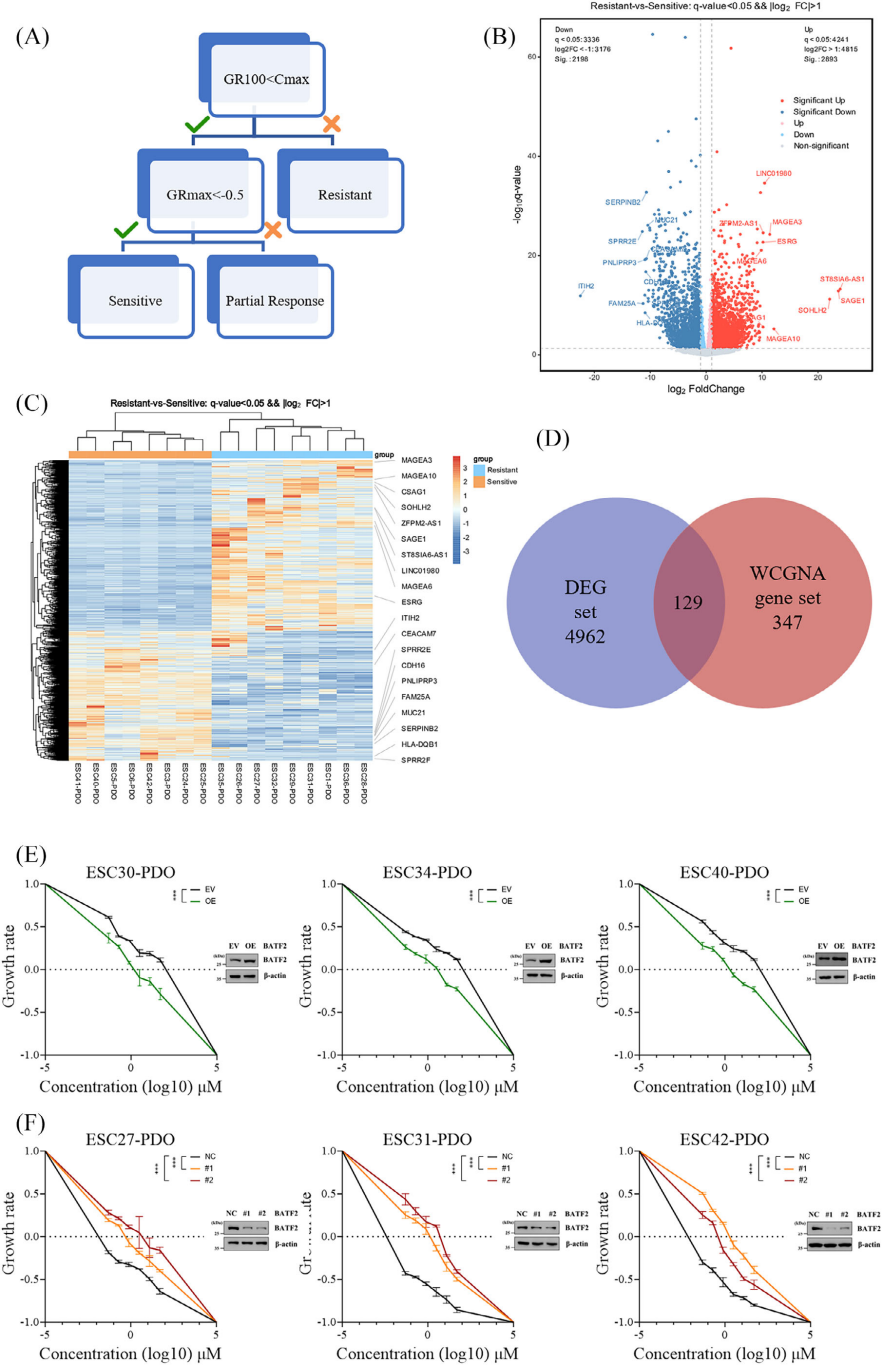
Identification of transcriptomic features associated with PTX resistance. (A) Decision tree integrating GR100, GRmax and *C*_max_ to classify PDO responses as PTX‐sensitive, partially responsive or resistant. (B) Volcano plot of differential gene expression between the PTX‐sensitive and PTX‐resistant groups. (C) Heatmap of DEG between PTX‐sensitive and PTX‐resistant groups. (D) Venn diagram displaying the overlap between the DEG set and the WGCNA gene set. (E) Dose–response curves of PTX upon BATF2 overexpression in three PTX‐resistant organoids (ESC30‐PDO, ESC34‐PDO and ESC40‐PDO). Data are from at least three independent experiments. Error bars represent means ± SD. ***, *p* < .001; EV, empty vector; OE, overexpression. (F) Dose–response curves of PTX upon BATF2 knockdown in three PTX‐sensitive organoids (ESC27‐PDO, ESC31‐PDO and ESC42‐PDO). Data are from at least three independent experiments. Error bars represent means ± SD. ***, *p* < .001. NC, negative control; #, sh BATF2.

Building on the reliability of the two‐step drug screening method, we next explore the transcriptional signatures of ESCC PDOs in response to PTX resistance to identify key biomarkers and underlying mechanisms. PTX is a classic anti‐tumour agent widely used in the treatment of various malignancies, including breast cancer,^50^ lung cancer,^51^ ovarian cancer^52^ and ESCC.^53^ In this study, PTX was used as an example to investigate its resistance mechanisms through PDO transcriptomic data. To refine the classification of drug response, we constructed a decision tree integrating GR100, GRmax and *C*_max_ values to distinguish PTX‐sensitive, partially responsive and resistant PDOs (Figure 6A). Nine PDOs (ESC6‐PDO, ESC21‐PDO, ESC22‐PDO, ESC29‐PDO, ESC30‐PDO, ESC33‐PDO, ESC34‐PDO, ESC36‐PDO and ESC40‐PDO) were identified as PTX‐resistant (GR100 > *C*_max_). Eight PDOs (ESC24‐PDO, ESC25‐PDO, ESC27‐PDO, ESC31‐PDO, ESC32‐PDO, ESC42‐PDO, ESC47‐PDO and ESC51‐PDO) were identified as PTX‐sensitive (GR100 < *C*_max_ and GRmax < –.5). Figure 6B displays a volcano plot of DEG, highlighting key genes that were significantly upregulated or downregulated. Figure 6C presents a heatmap of RNA‐Seq data from PTX‐sensitive and resistant groups, revealing distinct clusters that reflect specific gene expression differences between the two groups. KEGG pathway enrichment analysis of these genes identified several signalling pathways associated with PTX resistance (Figure S8), including ‘transcriptional misregulation in cancer’, ‘Wnt signalling pathway’, ‘TGF‐beta signalling’^54^, ^55^ and ‘Hippo signalling’.^56^, ^57^, ^58^

We further explored gene co‐expression patterns using WGCNA to identify gene modules potentially associated with PTX resistance. The power value used in this analysis was set to 28 (Figure S9A). To visualise the similarity between genes within each module, a gene network heatmap based on the Topological Overlap Matrix was constructed (Figure S9B). Four modules revealed significant correlation with PTX resistance (Figure S9C).

Subsequently, we performed an intersection analysis between DEG and WGCNA‐identified modules. A total of 129 genes (Figure 6D and Table S7) were significantly associated with resistance. Several genes, such as BATF2 and INPPL1, were significantly downregulated in the PTX‐resistant group. Overexpression of BATF2 in PTX‐resistant organoids (ESC30‐PDO, ESC34‐PDO and ESC40‐PDO) increased sensitivity to PTX treatment (Figure 6E). In contrast, knockdown of BATF2 in PTX‐sensitive organoids (ESC27‐PDO, ESC31‐PDO and ESC42‐PDO) decreased drug sensitivity (Figure 6F). Similar results were observed in the dose–response curves following overexpression (Figure S9D) and knockdown (Figure S9E) of INPPL1. These findings implicate the two genes as negative modulators of chemotherapy resistance, consistent with previous studies.^59^, ^60^

### 3.5 Dynamic DST using *C*_max_/GR100 offers alternative therapeutic options

**FIGURE 7:**
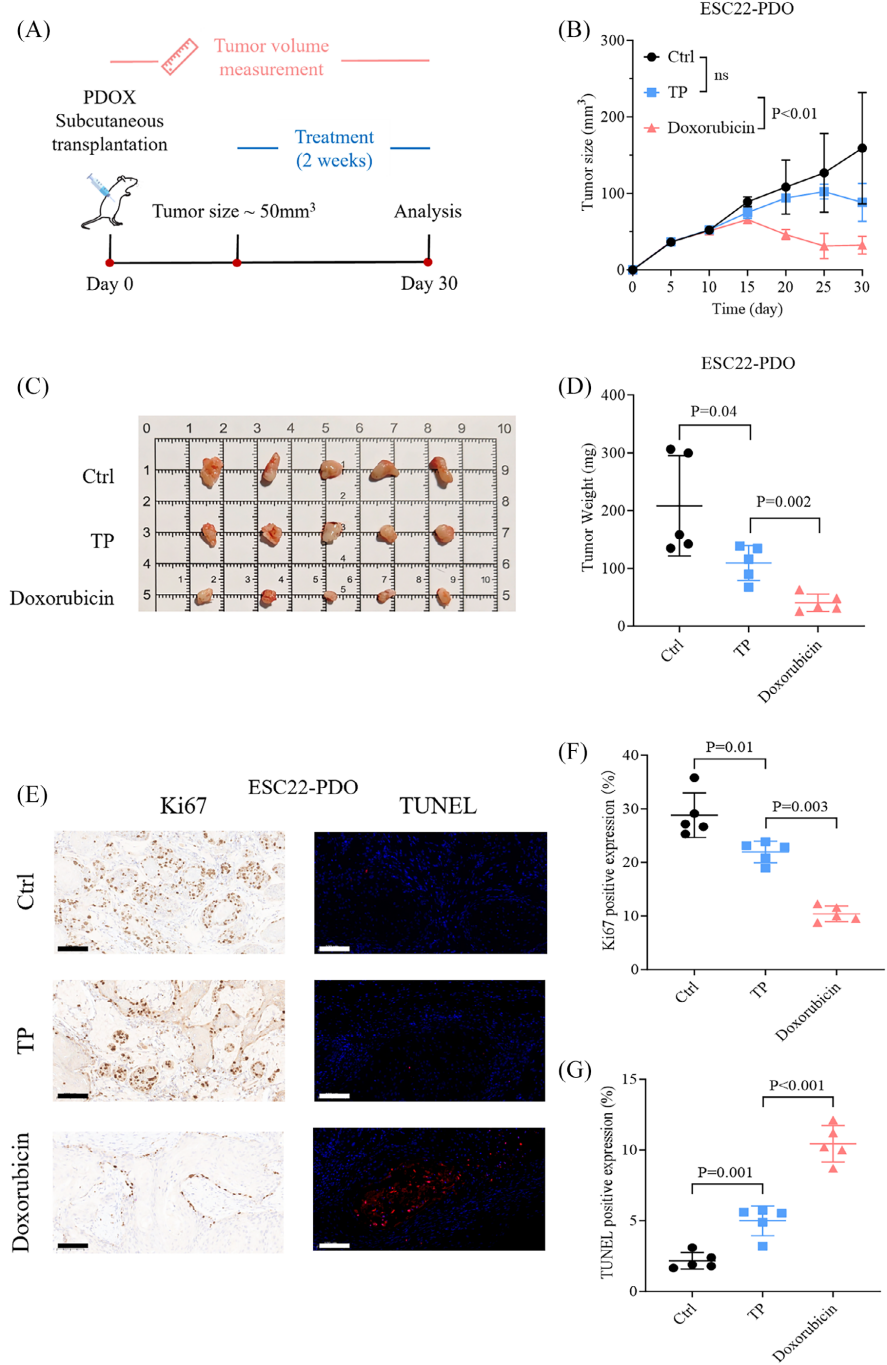
ESC22‐PDOX drug sensitivity assay process and results. (A) Experimental timeline and methodology for evaluating treatment efficacy in the PDOX model. Detailed procedures are described in *Methods* section. (B) Tumour volume changes over time in the ESC22‐PDOX treated with NC, TP and doxorubicin (*n* = 5, mean ± SD). (C) Tumour images at sacrifice. (D) Tumour weight at sacrifice (*n* = 5, mean ± SD). (E) Representative Ki‐67 IHC images and TUNEL immunofluorescence images. Scale bars, 100 µm. (F) Quantification of Ki‐67 IHC staining. (G) Quantification of TUNEL immunofluorescence staining. *Abbreviation*: PDOX, patient‐derived tumour organoid xenograft.

While organoid models hold considerable potential for identifying resistance targets and mechanisms, the immediate clinical priority is to develop pharmacologically‐based screening strategies that provide alternative treatment options for TP‐resistant patients. The classic pharmacokinetic parameter, *C*_max_/IC_50_, has been widely used to assess the targeted efficacy of drugs. A higher *C*_max_/IC_50_ ratio indicates stronger drug inhibition at therapeutic concentrations, potentially improving efficacy while reducing side effects.^61^, ^62^ Based on this concept, we hypothesise that the *C*_max_/GR100 ratio can also be used to assess the efficacy of different drugs and to screen for alternative therapies in TP‐resistant patients. Due to ethical considerations and the potential for increased animal sacrifice with multiple drug groups, we selected doxorubicin for in vivo validation, as it demonstrated significant efficacy in most drug‐resistant patients. ESC22‐PDO (NAT group) and ESC30‐PDO (treatment‐naïve group) were selected for in vivo validation due to their strong resistance profiles, reflected by the highest AUCsum values (Figure 3), indicating pronounced drug resistance. The *C*_max_/GR100 values for the drugs in these models were as follows: ESC22‐PDO (doxorubicin, 76.73; cisplatin, .07; PTX, .3; Table S5) and ESC30‐PDO (doxorubicin, 32.83; cisplatin, .05; PTX, .2; Table S5). Patient‐derived tumour organoid xenograft (PDOX) models were established following the protocols outlined in Figure 7A. In vivo drug sensitivity results for ESC22‐PDOX were consistent with in vitro screening. Compared with the NC group, the TP‐based chemotherapy group revealed some resistance: Terminal tumour weight differed significantly (TP vs. control, *p* = .04; Figure 7D), but no significant difference in tumour growth was observed (TP vs. control, ns; Figure 7B). In contrast, the doxorubicin group (highest *C*_max_/GR100) revealed a significant therapeutic response, with superior tumour inhibition compared with the TP group (tumour growth curve, TP vs. doxorubicin, *p *< .01; Figure 7B; terminal tumour weight, *p* = .002; Figure 7D). Similarly, in the ESC30‐PDOX model, the TP‐resistant phenotype was evident, with no significant therapeutic response in either tumour growth or terminal tumour weight (tumour growth curve, TP vs. control, ns; Figure S10A; terminal tumour weight, *p* = .11; Figure S10C). Doxorubicin, however, achieved greater tumour suppression than TP (tumour growth curve, TP vs. doxorubicin, *p *< .05; Figure S10A; terminal tumour weight, *p* = .003; Figure S10C).

IHC staining of ESC22‐PDOX and ESC30‐PDOX tumours confirmed positive expression of p40 and p63, validating the diagnosis of ESCC (Figure S11). TP treatment in ESC22‐PDOX led to a significant reduction in Ki67 positivity, indicating suppressed proliferation (Figure 7E), and increased TUNEL positivity, suggesting enhanced apoptosis (Figure 7E), with both changes reaching statistical significance (Ki67, TP vs. control, *p* = .01; Figure 7F; TUNEL, TP vs. control, *p* = .001; Figure 7G). Doxorubicin treatment revealed superior effects, with lower Ki67 positivity and stronger TUNEL expression (Ki67, TP vs. doxorubicin, *p* = .003; Figure 7F; TUNEL, TP vs. doxorubicin, *p* < .001; Figure 7G). In the ESC30‐PDOX model, TP treatment also suppressed proliferation and increased apoptosis (Ki67, TP vs. control, *p* = .005; Figure S10E; TUNEL, TP vs. control, *p* = .002; Figure S10F). Doxorubicin demonstrated greater efficacy, with stronger tumour inhibition and increased apoptosis compared with TP (Ki67, TP vs. doxorubicin, *p* < .001; Figure S10E; TUNEL, TP vs. doxorubicin, *p* = .009; Figure S10F). These findings suggest that although TP demonstrates anti‐tumour activity in resistant ESCC PDOX models, doxorubicin, selected through *C*_max_/GR100 screening, achieves stronger tumour suppression.

To further validate the robustness of the *C*_max_/GR100 metric, we tested A549 cells and their drug‐resistant derivatives (A549/DDP and A549/Tax) with PTX (Figure S12A) and cisplatin (Figure S12B). For both drugs, parental A549 cells showed higher *C*_max_/GR_100_ values than resistant lines, confirming that this parameter reliably indicates drug sensitivity and can be applied across models.

## 4 DISCUSSION

This study establishes one of the largest biobanks of patient‐derived organoid (PDO) models (*n* = 26) from locally advanced ESCC patients treated with perioperative chemotherapy. By including both treatment‐naïve and post‐chemotherapy organoids, our biobank provides a comprehensive representation of drug responses across diverse clinical stages, reflecting the clinical heterogeneity of ESCC. We developed an innovative two‐step drug screening method based on GR values, which was validated through its correlation with in vitro drug sensitivity and clinical outcomes. This approach significantly reduced the experimental timeline, enabling the early identification of drug‐resistant patients while preserving the genotypic and phenotypic stability of organoids by minimising genetic alterations from prolonged culture.^15^, ^63^ While previous studies have contributed significantly to elucidating resistance mechanisms in ESCC organoids, they have often overlooked the need for real‐time strategies to identify alternative treatments for resistant patients.^26^, ^27^ Our *C*_max_/GR100‐based screening method addresses this by enabling timely adjustments to treatment within the critical 30‐day window between surgery and subsequent cycles. These findings validate the method's ability to identify effective alternative drugs, facilitating therapy ‘repositioning’ and precise re‐treatment of resistant subgroups.

### 4.1 Clinically translatable drug screening procedure for broad laboratory adoption

While single‐dose drug screening is commonly used in cell line‐based assays, our study applies this approach to organoids, which provide drug sensitivity profiles more closely aligned with clinical tumour responses. We demonstrate that this method not only accurately reflects patient outcomes but also significantly shortens the drug screening timeline, enabling faster clinical decision‐making. In contrast to traditional cell lines, organoids offer a clinically relevant model that bridges the gap between laboratory findings and personalised therapy.

In drug screening, the scale of compound libraries typically correlates with the required cell culture volume, often necessitating extensive cell amplification and extended culture times before large‐scale screening. Previous studies have proposed strategies to balance compound numbers and screening timelines. For example, Tan et al.^64^ shortened the time from biopsy to drug screening by adjusting drug concentration windows and reducing dose‐titration points. In a cohort of 27 metastatic colorectal cancer patients, their two‐point titration method (requiring 144 000 cells) achieved similar results to the original nine‐point titration, providing outcomes for 68.8% of colorectal liver metastasis patients within 7 weeks. Similarly, Merrill et al.^65^ used Food and Drug Administration (US FDA)‐approved *C*_max_ to optimise drug screening in bladder cancer organoids, integrating multi‐omics data to identify predictive biomarkers. However, simplified screening methods (for example, two‐point titration^64^ or single‐point assays^65^) may have limitations for clinical application. Additionally, automated systems, such as the Tecan EVO 100 liquid handler, have enabled high‐throughput screening of over 1000 US FDA‐approved compounds, identifying effective agents for pancreatic cancer organoids.^66^ Despite their efficiency, these automated systems are costly and complex, limiting their accessibility for resource‐constrained labs. The two‐step drug screening method in this study eliminates the need for large‐scale cell amplification, providing a clinically relevant, cost‐effective alternative to traditional and automated screening methods. Furthermore, for laboratories with existing automated capabilities, our two‐step method can enhance screening efficiency by integrating with high‐throughput systems, thereby strengthening clinical translation capabilities.

In this study, we introduced *C*_max_/GR100 as a novel metric for evaluating drug efficacy, allowing precise identification of drugs with sustained effects at clinically achievable plasma concentrations. This was validated in vivo, where PDOX models with resistant strains (ESC22‐PDO and ESC30‐PDO) revealed resistance to TP combination therapy, consistent with in vitro results. In contrast, doxorubicin, which exhibited the highest *C*_max_/GR100 value, displayed a significant anti‐tumour effect. Doxorubicin, a broad‐spectrum anthracycline antibiotic, is widely used to treat various cancers, including breast, ovarian, leukaemia and lung cancers.^67^ Although not recommended as a first‐line treatment for ESCC in clinical guidelines,^68^, ^69^ doxorubicin has exhibited potential therapeutic efficacy in previous studies.^70^, ^71^ While this study demonstrated that doxorubicin may be effective in certain TP‐resistant ESCC cases, we do not recommend its universal use as a first‐line treatment. Instead, we propose a *C*_max_/GR100‐based drug screening strategy: Applying organoid‐based dynamic screening to identify alternative sensitive drugs for resistant patients, thereby providing a theoretical basis for ‘repurposing’ existing drugs such as doxorubicin and enabling precise re‐treatment for resistant subgroups.

### 4.2 Limitations of the study

This study has several limitations. For four patients (ESC5, ESC29, ESC40 and ESC51), organoid drug sensitivity results did not align with their clinical outcomes. Specifically, for ESC5 and ESC51, organoid drug screening indicated sensitivity to chemotherapy; however, the patient experienced disease progression in clinical practice. This discrepancy may be due to the organoid model's inability to fully replicate the tumour microenvironment (TME). Components of the TME, such as tumour‐associated fibroblasts, immune cells and stromal tissue, can significantly influence drug efficacy through modulation of drug resistance‐related signalling pathways.^72^, ^73^, ^74^, ^75^ Accordingly, although the organoid model suggested sensitivity, resistance mechanisms within the TME may have contributed to the patient's poor clinical response. In contrast, for ESC29 and ESC40, the organoid model predicted resistance, yet the patient achieved stable disease, likely due to the addition of immunotherapy, which was not assessed in the organoid model. AUCsum was used as a core predictor. However, considering the potential antagonistic or synergistic interactions between the drugs, its generalisability requires further validation with larger sample sizes. The toxic effects of doxorubicin were not evaluated in this study. Future research should further investigate its potential toxicities, which are essential for fully assessing its therapeutic potential. The drug library employed in this study may have limitations in its coverage, as it does not encompass all potentially relevant compounds. Furthermore, the lack of an immune microenvironment in the organoid cultures constrains the assessment of immunotherapy. The small sample size (*n* = 26), limited to patients with perioperative chemotherapy, may restrict the generalisability of these findings. To strengthen the reliability and broader applicability of our results, future studies should include larger sample sizes and multi‐centre trials to pinpoint the patient subset most likely to derive clinical benefit. Moreover, additional validation of the *C*_max_/GR100 values across a broader range of organoid lines is essential to confirm their robustness and reproducibility across diverse models.

## 5 CONCLUSIONS

In conclusion, the two‐step drug screening method based on GR values demonstrates significant clinical translational potential. By shortening evaluation time, strengthening clinical relevance and enabling direct comparisons across drugs through the *C*_max_/GR100 method, this approach facilitates the rapid identification of drug‐resistant patients and the selection of alternative therapies. It offers an innovative strategy for personalised ESCC treatment, with scope for further optimisation and broader application to other cancer types.

## AUTHOR CONTRIBUTIONS

C.Y.S., S.B.Q. and J.Z. designed the experiments and interpreted the data. C.Y.S., S.J. and X.T. conducted experiments and developed methodologies. C.Y.S., S.J., Y.L., Y.X.Z., J.Y., K.H., C.L., Y.C., W.J.D. and J.Z. managed ESCC tumour samples. C.Y.S., X.T., Y.F.X. and C.D. acquired experimental data. C.Y.S., Y.C. and C.D. wrote the manuscript. C.D., S.B.Q., J.Z., S.J., Y.C., W.J.D. and C.L. provided administrative, technical or material support. C.Y.S. and J.Z. have accessed and verified the underlying data and C.Y.S. and J.Z. were responsible for the decision to submit the manuscript. All authors approved the manuscript.

## CONFLICT OF INTERESTS STATEMENT

The authors declare no competing interests.

## ETHIC STATEMENT

This study was performed in line with the principles of the Declaration of Helsinki. Approval was granted by the Ethics Committee of the First Affiliated Hospital of Soochow University.

## CONSENT

All samples were collected following written informed consent from the patients and in compliance with the ethical standards outlined in the Declaration of Helsinki. All animal experiments were approved and monitored by the Center of Experimental Animals of Soochow University (Animal Welfare Assurance No. 202408A0242).

## Supporting information



Table S1. Excel file containing additional data too large to fit in a PDF, related to Methods.

Table S2. Excel file containing even more data too large to fit in a PDF, related to Figure 1.

Document S1. Figures S1–S12 and Tables S3–S7

## Data Availability

This study generated a biobank of organoids derived from ESCC patients. All primary data associated with this study are available upon request to the corresponding author for individuals with appropriate data‐sharing agreements in place. All data related to this study are present in the paper or Supplementary Materials.
