# Supplementary material for: Organoid‐based two‐step drug screening for rapid identification of chemotherapy‐resistant oesophageal squamous cell carcinoma and alternative therapies
Source: Clin Transl Med. 2025 Nov 20;15(11):e70534. doi: 10.1002/ctm2.70534 (PMC12635425; doi:10.1002/ctm2.70534)

Supplemental information

Title:

Organoid-Based Two-Step Drug Screening Method for Rapidly Identifying Chemotherapy-Resistant Esophageal Squamous Cell Carcinoma Patients and Alternative Therapies

Authors:

Chen-ye Shao [1, 2, #], *Sheng Ju* [1, 2, #], *Xin Tong* [1, 2, #], *Kang Hu* [1, 2, #], *Yu Li* [1, 2], *Yi-xian Zhu* [3], *Jian Yang* [1, 2], *Chang Li* [1, 2], *Yu-feng Xie* [1, 2], *Yuan Cui* [1, 2], *Wei-jun Deng* [1, 2], *Cheng Ding* [1, 2, ***], *Song-bing Qin* [3, **], *and Jun Zhao* [1, 2, *]

Figure S1 Genomic mutation profiles and the transcriptomic consistency analysis of PDOs and matched tumor tissues, related to Figure 2. (**A**) The genomic mutation profiles of PDOs and their matched tumor tissues are displayed. Red : Frameshift mutations; Orange: Nonsense mutations; Blue: Missense mutations; Yellow: In-frame insertions/deletions. (**B**) A correlation matrix showing the transcriptomic consistency between PDOs and matched tumor tissues.

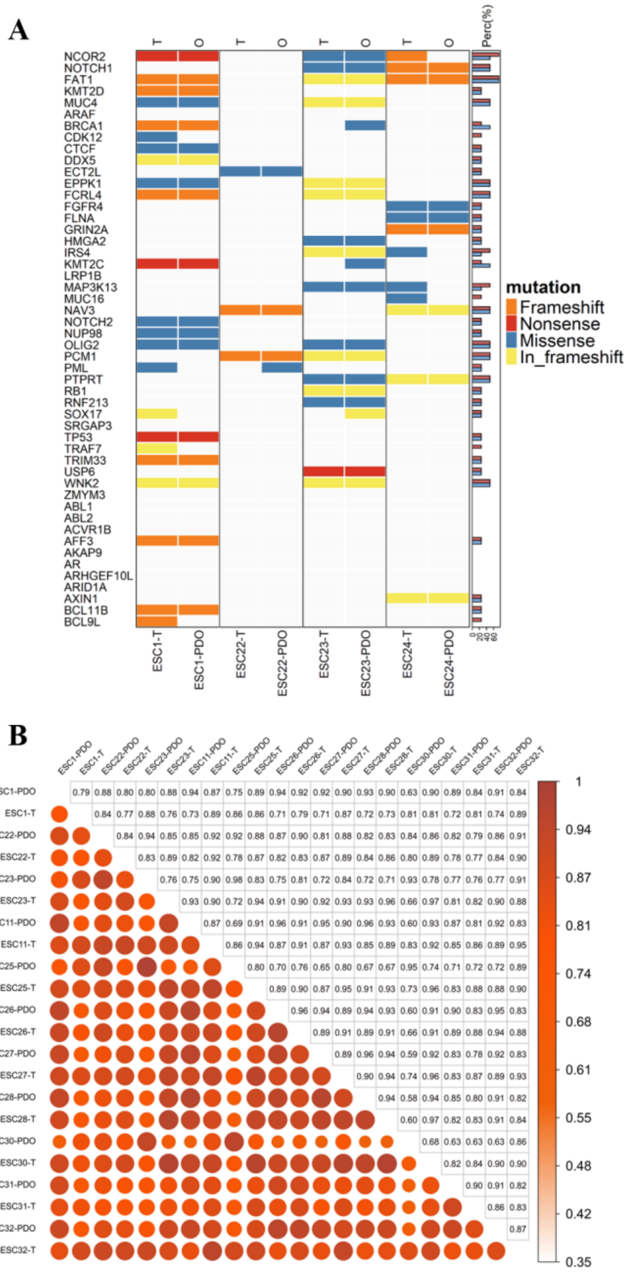

Table S3 RNA expression correlation analysis between PDOs and their source tumor tissues, related to Figure 2. This table presents the results of the RNA expression correlation analysis between PDOs and their matched tumor tissues. Data were calculated using the Pearson Correlation Coefficient (PCC), reflecting the transcriptomic similarity between each organoid and its matched tumor tissue. The RNA expression correlation value between organoids and tumor tissues, ranging from 0 to 1, with values closer to 1 indicating higher expression similarity.

Sample	RNA Expression Correlation
ESC1-PDO vs T	0.80
ESC22-PDO vs T	0.85
ESC23-PDO vs T	0.77
ESC11-PDO vs T	0.88
ESC25-PDO vs T	0.80
ESC26-PDO vs T	0.97
ESC27-PDO vs T	0.90
ESC28-PDO vs T	0.95
ESC30-PDO vs T	0.69
ESC31-PDO vs T	0.91
ESC32-PDO vs T	0.87

Figure S2 Heatmap summarizing the preliminary drug screening results across different ESCC organoid models, related to Figure 3. Each organoid was screened with 10 different compounds (specific compound information is provided in **Table 1**). The color scale represents cell viability, with red indicating poor drug efficacy (high viability) and blue indicating strong drug efficacy (low viability).

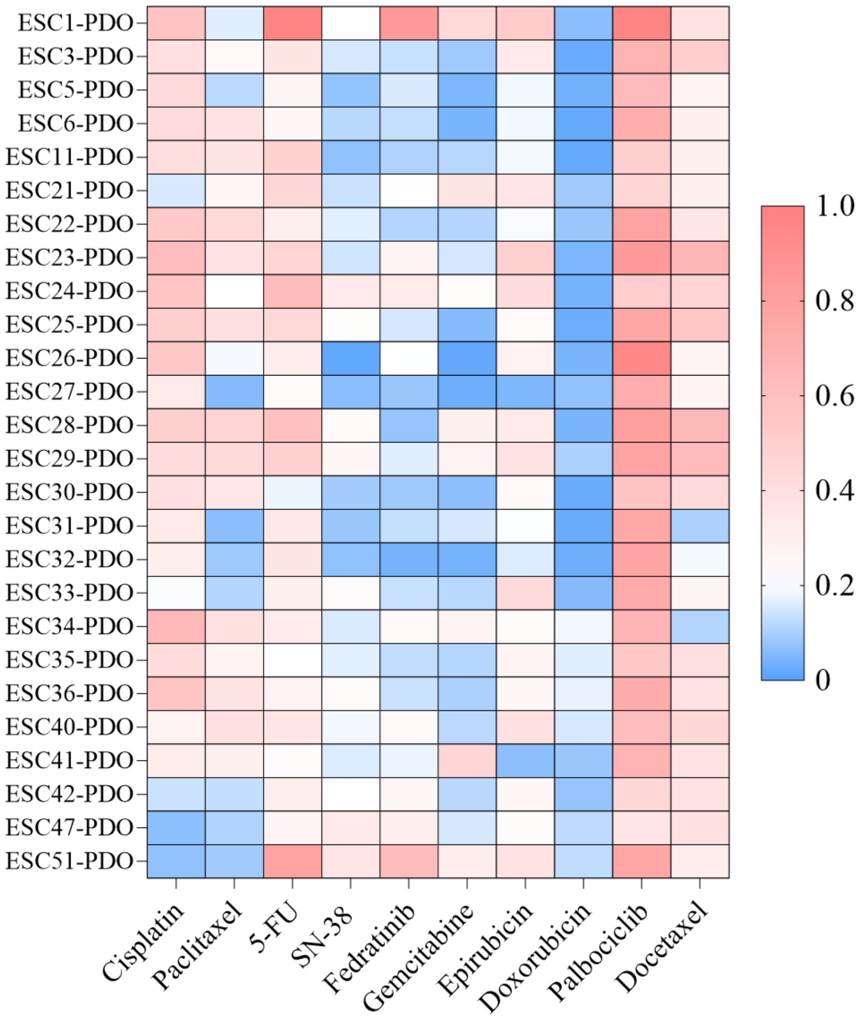

Table S4 Summary of preliminary screening results for different organoid models, related to Figure 3. Drugs with the most effective inhibition (selection criteria: cell viability ≤ 20%, and the top three most inhibitory drugs) were selected for secondary screening

Cell line		Drugs									
		Cisplatin	Paclitaxel	5-FU	SN-38	Fedratinib	Gemcitabine	Epirubicin	Doxorubicin	Palbociclib	Docetaxel
ESC1-PDO	Mean	0.57	0.16	0.97	0.20	0.84	0.44	0.53	0.06	0.96	0.38
	SD	0.027	0.018	0.042	0.012	0.050	0.020	0.037	0.008	0.022	0.036
ESC3-PDO	Mean	0.40	0.24	0.37	0.15	0.13	0.09	0.34	0.02	0.67	0.51
	SD	0.024	0.030	0.066	0.006	0.012	0.010	0.017	0.007	0.006	0.015
ESC5-PDO	Mean	0.43	0.12	0.27	0.07	0.16	0.05	0.18	0.03	0.63	0.26
	SD	0.110	0.006	0.016	0.008	0.007	0.001	0.059	0.007	0.065	0.037
ESC6-PDO	Mean	0.43	0.37	0.26	0.12	0.13	0.04	0.18	0.02	0.71	0.30
	SD	0.101	0.105	0.002	0.015	0.013	0.016	0.008	0.005	0.141	0.044
ESC11-PDO	Mean	0.40	0.37	0.48	0.07	0.11	0.11	0.19	0.02	0.51	0.29
	SD	0.015	0.044	0.021	0.003	0.012	0.011	0.014	0.003	0.029	0.020
ESC21-PDO	Mean	0.15	0.26	0.45	0.14	0.21	0.36	0.36	0.09	0.46	0.30
	SD	0.028	0.034	0.040	0.039	0.042	0.040	0.031	0.024	0.053	0.021
ESC22-PDO	Mean	0.54	0.44	0.30	0.16	0.11	0.11	0.19	0.08	0.78	0.35
	SD	0.024	0.026	0.039	0.018	0.009	0.012	0.009	0.012	0.036	0.032
ESC23-PDO	Mean	0.62	0.38	0.47	0.14	0.27	0.15	0.49	0.04	0.83	0.66
	SD	0.080	0.033	0.052	0.012	0.028	0.011	0.004	0.006	0.055	0.065
ESC24-PDO	Mean	0.56	0.21	0.62	0.32	0.33	0.21	0.41	0.04	0.51	0.47
	SD	0.036	0.033	0.023	0.051	0.036	0.044	0.150	0.008	0.092	0.140
ESC25-PDO	Mean	0.50	0.39	0.44	0.22	0.15	0.06	0.23	0.03	0.76	0.56
	SD	0.043	0.006	0.048	0.026	0.037	0.004	0.017	0.004	0.007	0.109
ESC26-PDO	Mean	0.54	0.19	0.32	0.02	0.20	0.02	0.29	0.04	0.94	0.26
	SD	0.020	0.005	0.021	0.003	0.015	0.002	0.042	0.006	0.046	0.014
ESC27-PDO	Mean	0.33	0.06	0.22	0.06	0.08	0.03	0.05	0.07	0.71	0.26
	SD	0.036	0.005	0.023	0.004	0.007	0.003	0.006	0.010	0.029	0.037
ESC28-PDO	Mean	0.50	0.47	0.59	0.23	0.08	0.29	0.33	0.04	0.80	0.65
	SD	0.030	0.045	0.021	0.013	0.014	0.027	0.026	0.007	0.119	0.034
ESC29-PDO	Mean	0.41	0.43	0.49	0.25	0.16	0.29	0.38	0.10	0.77	0.63
	SD	0.038	0.049	0.021	0.046	0.036	0.071	0.013	0.026	0.076	0.042
ESC30-PDO	Mean	0.39	0.35	0.18	0.09	0.09	0.07	0.23	0.02	0.58	0.43
	SD	0.033	0.063	0.023	0.027	0.016	0.005	0.015	0.004	0.109	0.020

ESC31-PDO	Mean	0.34	0.06	0.34	0.08	0.13	0.15	0.20	0.02	0.75	0.10
	SD	0.014	0.006	0.016	0.007	0.004	0.024	0.016	0.005	0.066	0.024
ESC32-PDO	Mean	0.30	0.09	0.37	0.07	0.04	0.04	0.16	0.03	0.76	0.19
	SD	0.038	0.006	0.048	0.005	0.003	0.007	0.005	0.004	0.193	0.030
ESC33-PDO	Mean	0.19	0.11	0.29	0.23	0.13	0.12	0.43	0.06	0.73	0.27
	SD	0.008	0.023	0.012	0.035	0.010	0.021	0.074	0.024	0.075	0.066
ESC34-PDO	Mean	0.64	0.39	0.32	0.16	0.24	0.27	0.22	0.18	0.66	0.11
	SD	0.037	0.042	0.029	0.021	0.040	0.029	0.029	0.012	0.085	0.024
ESC35-PDO	Mean	0.42	0.28	0.20	0.16	0.13	0.11	0.27	0.16	0.54	0.40
	SD	0.017	0.019	0.033	0.010	0.025	0.022	0.018	0.033	0.068	0.016
ESC36-PDO	Mean	0.57	0.37	0.29	0.23	0.14	0.10	0.26	0.17	0.72	0.37
	SD	0.085	0.017	0.019	0.025	0.032	0.019	0.014	0.014	0.041	0.029
ESC40-PDO	Mean	0.28	0.39	0.36	0.18	0.23	0.12	0.38	0.15	0.62	0.45
	SD	0.022	0.065	0.045	0.017	0.012	0.011	0.019	0.032	0.047	0.011
ESC41-PDO	Mean	0.31	0.29	0.22	0.16	0.18	0.46	0.06	0.08	0.67	0.38
	SD	0.013	0.034	0.032	0.044	0.010	0.075	0.010	0.013	0.030	0.052
ESC42-PDO	Mean	0.14	0.13	0.30	0.20	0.24	0.12	0.25	0.08	0.45	0.38
	SD	0.008	0.033	0.016	0.012	0.021	0.020	0.043	0.019	0.099	0.036
ESC47-PDO	Mean	0.06	0.11	0.27	0.34	0.29	0.15	0.22	0.12	0.36	0.39
	SD	0.027	0.023	0.027	0.045	0.061	0.018	0.014	0.022	0.035	0.015
ESC51-PDO	Mean	0.07	0.09	0.78	0.36	0.62	0.32	0.38	0.12	0.75	0.31
	SD	0.015	0.022	0.094	0.036	0.072	0.043	0.074	0.021	0.114	0.050

Table S5 Summary of secondary screening results for different organoid models using the two-step drug screening method, related to Figure 3. Cmax, Maximum Concentration.

**Cell Line**	**Secondary screen**				**TP chemotherapy regimen**	
ESC1-PDO	Drugs	SN-38		Doxorubicin	Cisplatin	Paclitaxel
	GR100 (μM)	0.02		0.05	0.92	0.76
	Cmax/GR100	8.52		139.96	14.91	6.70
ESC3-PDO	Drugs	SN-38	Gemcitabine	Doxorubicin	Cisplatin	Paclitaxel
	GR100 (μM)	0.08	22.23	0.08	1.21	1.39
	Cmax/GR100	1.85	4.54	90.66	11.33	3.66
ESC5-PDO	Drugs	SN-38	Gemcitabine	Doxorubicin	Cisplatin	Paclitaxel
	GR100 (μM)	0.07	38.61	0.07	3.32	4.65
	Cmax/GR100	2.11	2.62	97.72	4.12	1.10
ESC6-PDO	Drugs (μM)	SN-38	Gemcitabine	Doxorubicin	Cisplatin	Paclitaxel
	GR100	0.09	10.26	0.10	1.65	7.96
	Cmax/GR100	1.71	9.84	66.22	8.26	0.64
ESC11-PDO	Drugs	SN-38	Fedratinib	Doxorubicin	Cisplatin	Paclitaxel
	GR100 (μM)	0.02	1.77	0.02	103.7	3.15
	Cmax/GR100	8.04	1.94	313.64	0.13	1.62
ESC21-PDO	Drugs	SN-38		Doxorubicin	Cisplatin	Paclitaxel
	GR100 (μM)	1.01		4.15	9.53	8.42
	Cmax/GR100	0.15		1.66	1.43	0.61
ESC22-PDO	Drugs	Fedratinib	Gemcitabine	Doxorubicin	Cisplatin	Paclitaxel
	GR100 (μM)	5.68	115.7	0.09	204.70	17.20
	Cmax/GR100	0.61	0.87	76.73	0.07	0.30
ESC23-PDO	Drugs	SN-38	Gemcitabine	Doxorubicin	Cisplatin	Paclitaxel
	GR100 (μM)	0.06	6.87	0.10	165.20	1.31
	Cmax/GR100	2.63	14.70	69.30	0.08	3.91
ESC24-PDO	Drugs			Doxorubicin	Cisplatin	Paclitaxel
	GR100 (μM)			0.03	0.05	0.04
	Cmax/GR100			228.70	276.11	118.14
ESC25-PDO	Drugs	Fedratinib	Gemcitabine	Doxorubicin	Cisplatin	Paclitaxel
	GR100 (μM)	0.35	14.45	0.03	9.79	0.83
	Cmax/GR100	9.93	6.99	259.50	1.40	6.16

ESC26-PDO	Drugs	SN-38	Gemcitabine	Doxorubicin	Cisplatin	Paclitaxel
	GR100 (μM)	0.01	0.63	0.05	9.15	2.02
	Cmax/GR100	37.13	159.23	135.88	1.49	2.52
ESC27-PDO	Drugs		Gemcitabine	Epirubicin	Cisplatin	Paclitaxel
	GR100 (μM)		0.23	0.04	0.04	0.04
	Cmax/GR100		439.90	4.47	310.26	132.02
ESC28-PDO	Drugs	SN-38	Fedratinib	Doxorubicin	Cisplatin	Paclitaxel
	GR100 (μM)	0.08	1.44	0.09	22.28	4.26
	Cmax/GR100	1.83	2.40	78.52	0.61	1.20
ESC29-PDO	Drugs		Fedratinib	Doxorubicin	Cisplatin	Paclitaxel
	GR100 (μM)		0.72	0.61	19.15	5.39
	Cmax/GR100		4.78	11.31	0.71	0.95
ESC30-PDO	Drugs	Fedratinib	Gemcitabine	Doxorubicin	Cisplatin	Paclitaxel
	GR100 (μM)	2.12	5.27	0.21	301.6	25.86
	Cmax/GR100	1.63	19.18	32.83	0.05	0.20
ESC31-PDO	Drugs	SN-38		Doxorubicin	Cisplatin	Paclitaxel
	GR100 (μM)	0.01		0.02	0.03	0.01
	Cmax/GR100	18.56		331.73	419.84	652.93
ESC32-PDO	Drugs	Fedratinib	Gemcitabine	Doxorubicin	Cisplatin	Paclitaxel
	GR100 (μM)	0.31	0.35	0.05	0.07	0.09
	Cmax/GR100	11.24	291.74	132.23	185.43	54.39
ESC33-PDO	Drugs		Gemcitabine	Doxorubicin	Cisplatin	Paclitaxel
	GR100 (μM)		15.84	0.56	6.76	5.12
	Cmax/GR100		6.38	12.32	2.02	1
ESC34-PDO	Drugs	SN-38	Doxorubicin	Docetaxel	Cisplatin	Paclitaxel
	GR100 (μM)	0.03	0.22	0.02	118.6	22.76
	Cmax/GR100	5	31.36	149	0.12	0.22
ESC35-PDO	Drugs	SN-38	Fedratinib	Gemcitabine	Cisplatin	Paclitaxel
	GR100 (μM)	0.03	0.26	6.97	36.06	3.14
	Cmax/GR100	5	13.23	14.49	0.38	1.63
ESC36-PDO	Drugs	Fedratinib	Gemcitabine	Doxorubicin	Cisplatin	Paclitaxel
	GR100 (μM)	0.51	10.07	0.11	58.41	5.13
	Cmax/GR100	6.75	10.04	62.73	0.23	0.99
ESC40-PDO	Drugs	SN-38	Gemcitabine	Doxorubicin	Cisplatin	Paclitaxel

	GR100 (μM)	0.09	15.33	0.77	12.16	28.75
	Cmax/GR100	1.67	6.59	8.96	1.12	0.18
ESC41-PDO	Drugs	SN-38	Epirubicin	Doxorubicin	Cisplatin	Paclitaxel
	GR100 (μM)	0.05	0.03	0.24	3.73	3.73
	Cmax/GR100	3	5.33	28.75	3.66	1.37
ESC42-PDO	Drugs		Gemcitabine	Doxorubicin	Cisplatin	Paclitaxel
	GR100 (μM)		1.73	0.12	1.1	0.02
	Cmax/GR100		58.4	57.5	12.48	330.74
ESC47-PDO	Drugs			Doxorubicin	Cisplatin	Paclitaxel
	GR100 (μM)			0.22	0.1	0.05
	Cmax/GR100			31.36	139.35	108.33
ESC51-PDO	Drugs			Doxorubicin	Cisplatin	Paclitaxel
	GR100 (μM)			0.13	0.29	0.04
	Cmax/GR100			53.07	46.85	133.4

Figure S3 Analysis of the drug response to Cisplatin in ESCC organoids derived from different patients, related to Figure 3. (**A**) Dose-response curves of different PDO cell lines treated with Cisplatin. Each curve represents the response of a PDO cell line to the drug (see legend for details). (**B**) Violin plot showing the distribution of normalized area under the curve (AUC) values for Cisplatin response. (**C**) Violin plot showing the distribution of log10-transformed GR100 values for Cisplatin response. (**D**) Correlation analysis between normalized AUC values and log10(GR100) values.

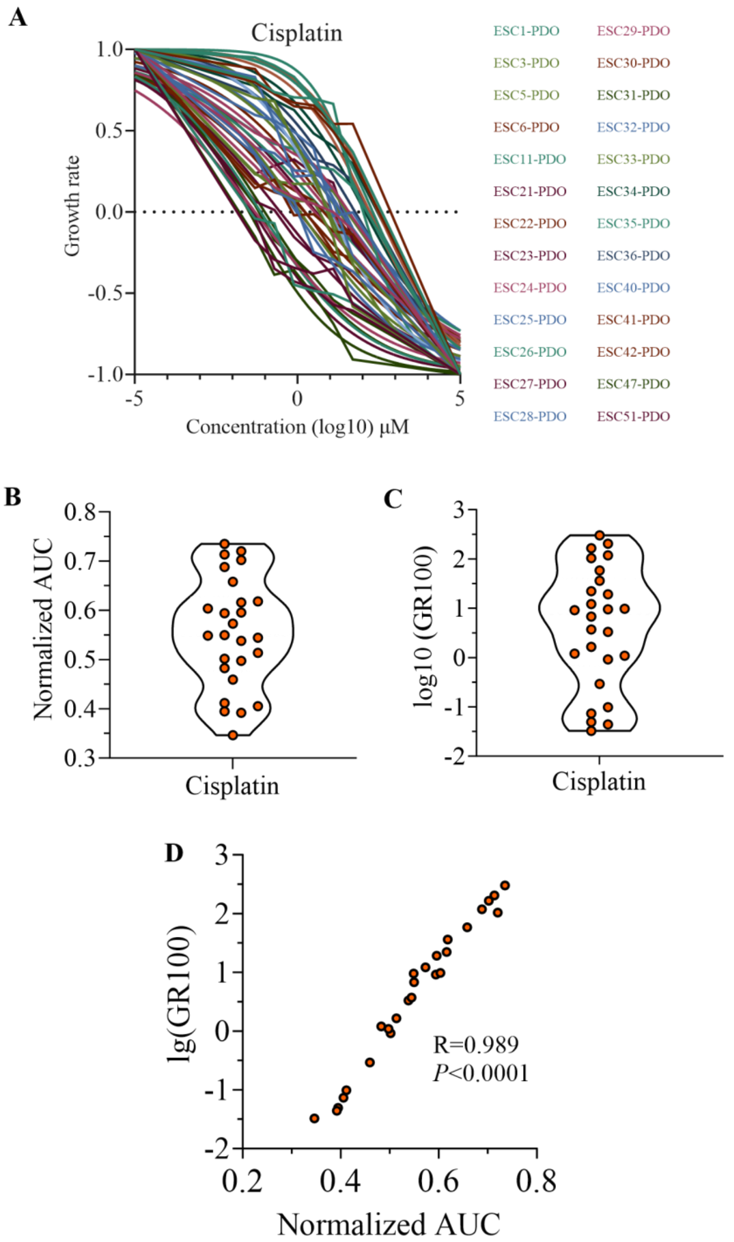

Figure S4 Analysis of the drug response to Paclitaxel in ESCC organoids derived from different patients, related to Figure 3. (**A**) Dose-response curves of different PDO cell lines treated with Paclitaxel. Each curve represents the response of a PDO cell line to the drug (see legend for details). (**B**) Violin plot showing the distribution of normalized area under the curve (AUC) values for Paclitaxel response. (**C**) Violin plot showing the distribution of log10-transformed GR100 values for Paclitaxel response. (**D**) Correlation analysis between normalized AUC values and log10(GR100) values.

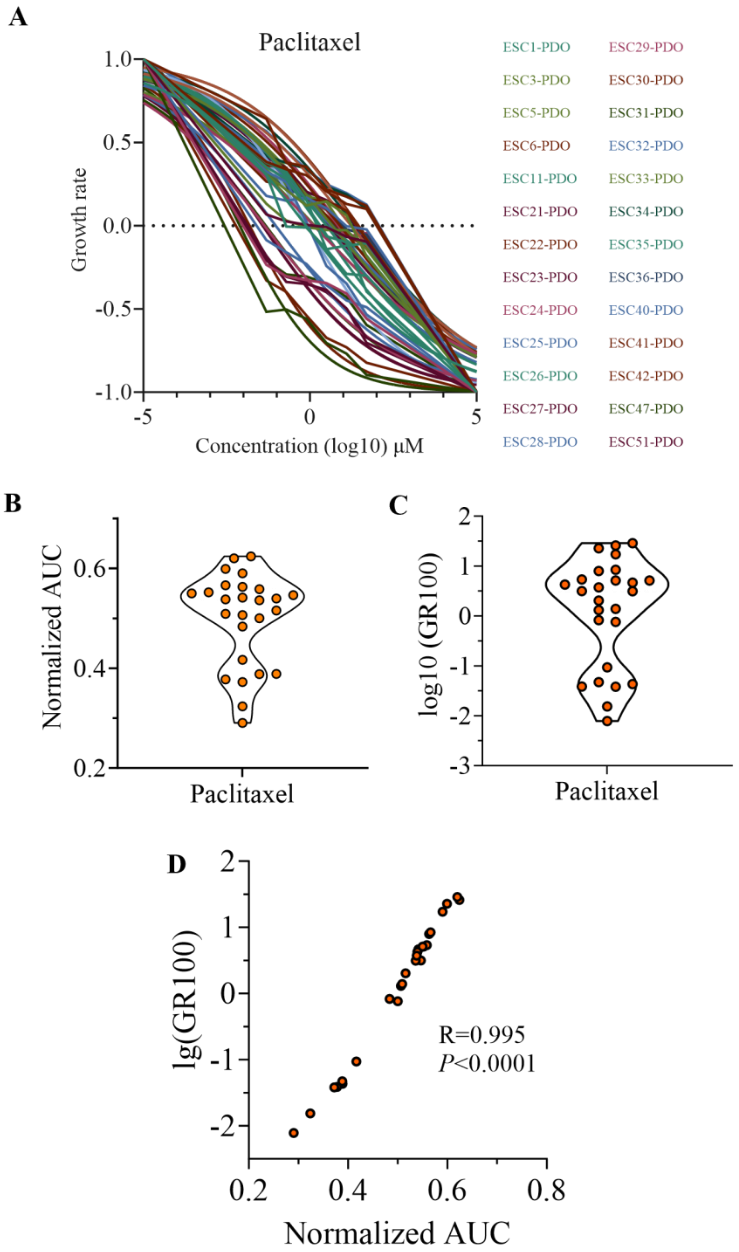

Figure S5 | Principal component analysis (PCA) and distribution of patient subgroups.

(**A**) PCA plot showing the distribution of treatment-naive group and NAT group. (**B**) PCA plot showing the distribution based on AUCsum. (**C**) Comparison of overall drug sensitivity between patient subgroups. No significant difference was observed between the two groups (ns, not significant; Fisher's exact test). NAT, neoadjuvant therapy.

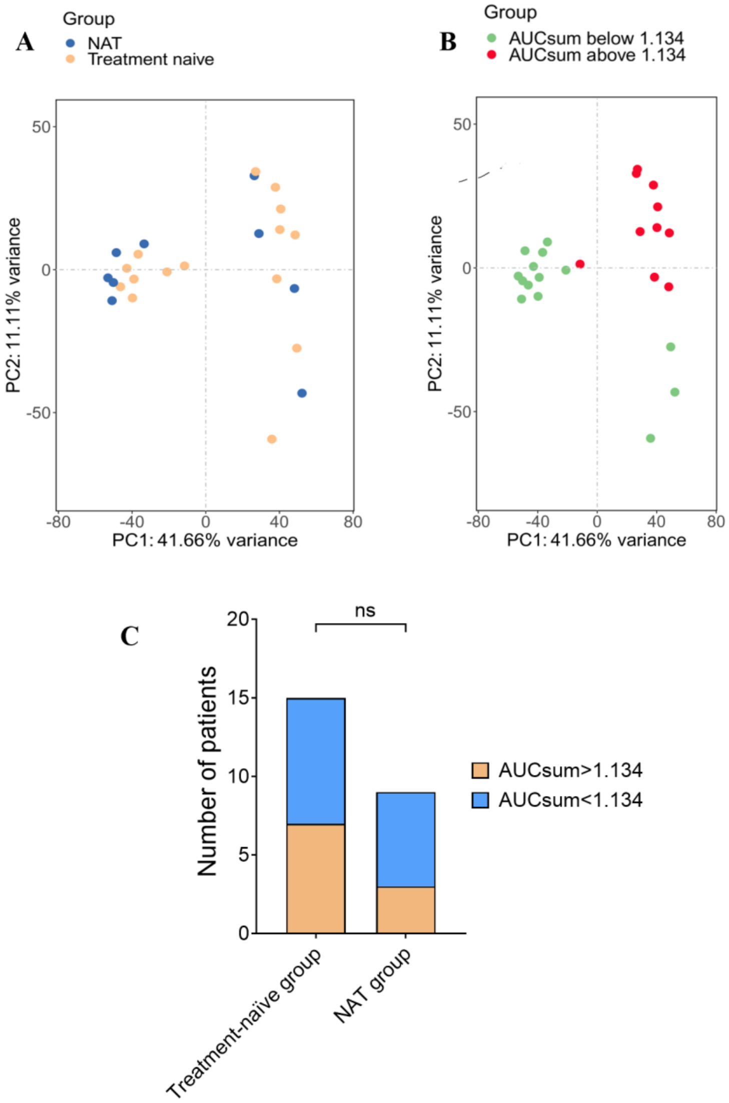

Figure S6 Treatment course, imaging evaluation before and after neoadjuvant therapy, and organoid drug sensitivity screening experiment of the ESC27 patient, related to Figure 4. (**A**) Treatment timeline of the ESC27 patient. (**B**) CT imaging comparison of the ESC27 patient before and after neoadjuvant therapy. The tumor location is indicated by the red arrow. Scale bars are 10 cm. (**C**) Preliminary drug screening results of ESC27-PDO. Scale bars are 50 µm. (**D**) Secondary drug sensitivity screening results of ESC27-PDO, showing the compounds Cisplatin, Paclitaxel, and Doxorubicin (drugs with the highest Cmax/GR100 values). Scale bars are 50 µm.

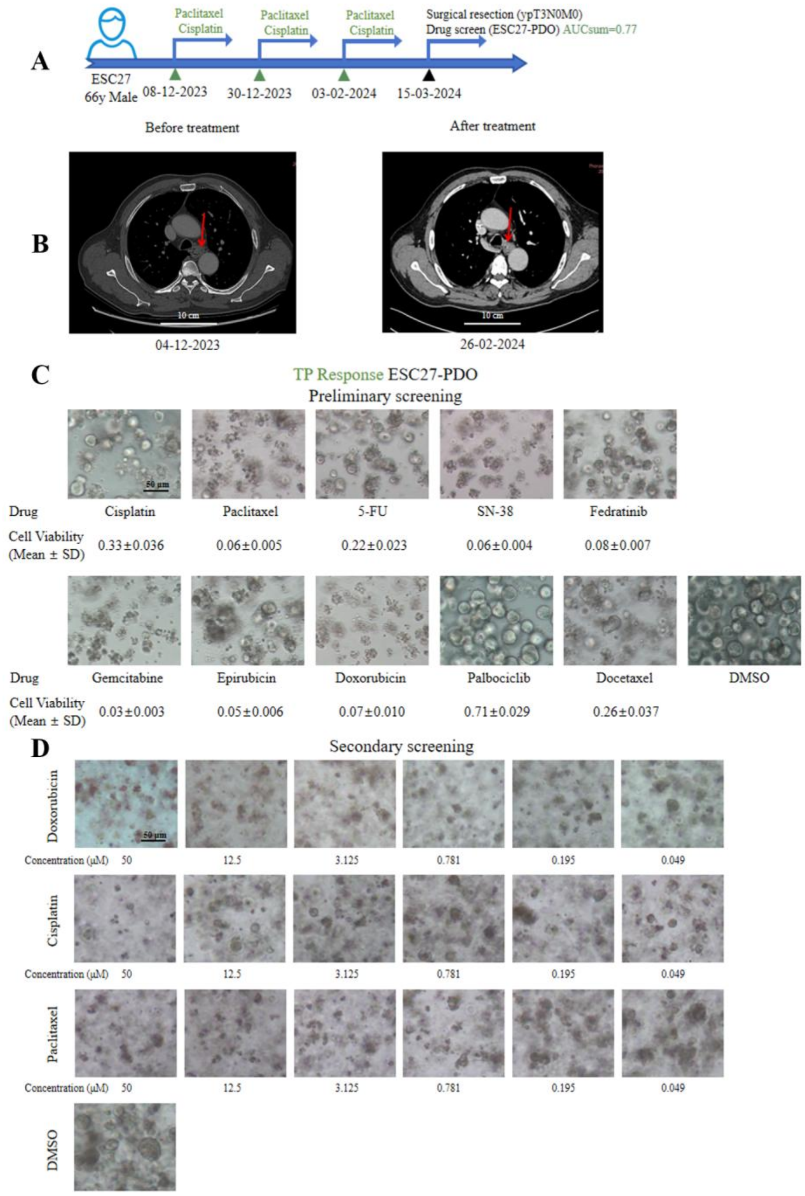

Figure S7 Apoptosis analysis of resistant (ESC23-PDO) and sensitive (ESC27-PDO) strains under treatment with Cisplatin, Paclitaxel, and DMSO, related to Figure 4. (**A**) Apoptosis was marked using Caspase 3/7 activity dye (green fluorescence). Representative fluorescence images of ESC23-PDO and ESC27-PDO on the second and fourth days following treatment with Cisplatin (1 μM), Paclitaxel (1 μM), and DMSO. Scale bars are 100 μm. (**B**) Relative green Caspase 3/7 signal intensity of ESC23-PDO and ESC27-PDO under Cisplatin and Paclitaxel treatment, normalized to the DMSO group.

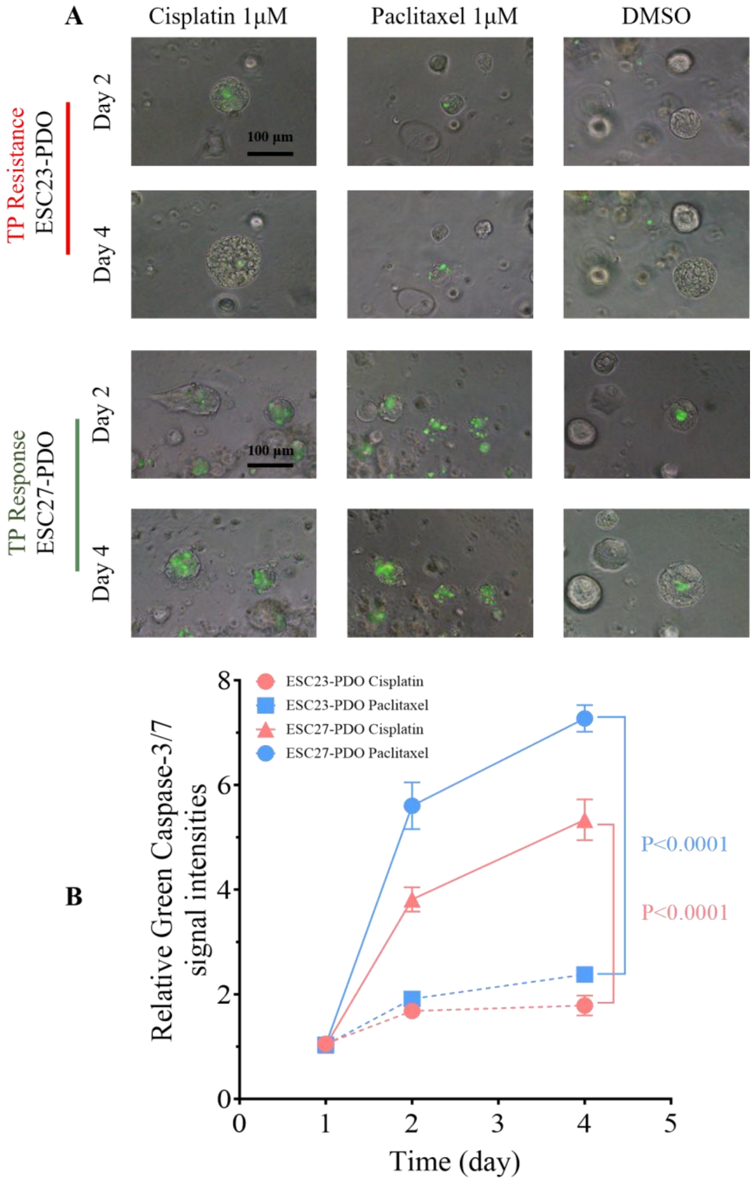

Table S6 Summary of results for 5 organoid models (ESC6-PDO, ESC23-PDO, ESC24-PDO, ESC27-PDO, ESC28-PDO, ESC35-PDO, ESC40-PDO, and ESC41-PDO) using the conventional drug screening method, related to Figure 5.

		ESC6 -PDO	ESC23 -PDO	ESC24 -PDO	ESC27 -PDO	ESC28 -PDO	ESC35 -PDO	ESC40 -PDO	ESC41 -PDO
Cisplatin	GR100 (μM)	2.80	206.20	0.09	0.08	43.12	33.46	16.21	2.88
	Cmax/GR100	4.89	0.07	144.79	181.40	0.32	0.41	0.84	4.75
Paclitacel	GR100 (μM)	16.58	1.75	0.28	0.07	6.80	4.46	30.23	4.42
	Cmax/GR100	0.31	2.92	18.29	76.37	0.75	1.14	0.17	1.15
5-FU	GR100 (μM)	26.01	44.67	34.68	29.82	105.90	8.78	31.25	3.37
	Cmax/GR100	0.29	0.17	0.22	0.25	0.07	0.85	0.24	2.23
SN-38	GR100 (μM)	0.04	0.09	0.09	0.09	0.16	0.18	0.17	0.17
	Cmax/GR100	3.38	1.76	1.71	1.76	0.95	0.83	0.88	0.88
Fedratinib	GR100 (μM)	1.34	1.83	1.60	2.26	1.41	0.88	3.11	2.11
	Cmax/GR100	2.56	1.88	2.15	1.52	2.44	3.91	1.11	1.63
Gemcitabine	GR100 (μM)	2.98	8.11	16.39	0.44	46.75	8.18	16.89	25.16
	Cmax/GR100	33.94	3.68	6.16	227.58	2.16	12.35	5.98	4.02
Epirubicin	GR100 (μM)	0.13	0.11	0.08	0.05	0.19	0.45	0.59	0.09
	Cmax/GR100	1.24	1.42	1.99	3.30	0.85	0.36	0.27	1.78
Doxorubicin	GR100 (μM)	0.06	0.06	0.01	0.04	0.08	0.88	1.12	0.78
	Cmax/GR100	113.86	109.09	497.48	175.22	88.65	7.84	6.16	8.85
Palbociclib	GR100 (μM)	3.86	29.76	8.53	15.10	26.85	27.88	29.12	30.12
	Cmax/GR100	0.06	0.01	0.03	0.01	0.01	0.01	0.01	0.01
Docetaxel	GR100 (μM)	2.56	7.61	10.96	11.04	22.55	27.96	24.53	26.77
	Cmax/GR100	1.16	0.39	0.27	0.27	0.13	0.11	0.12	0.11

Figure S8 KEGG pathway enrichment analysis bubble plot of differentially expressed genes, related to Figure 6. The size of the bubbles indicates the number of differentially expressed genes enriched in the pathway (GeneNumber), with larger bubbles corresponding to a higher number of differentially expressed genes. The color of the bubbles ranges from blue to red, representing the p-value significance level, with red indicating stronger significance and blue indicating lower significance.

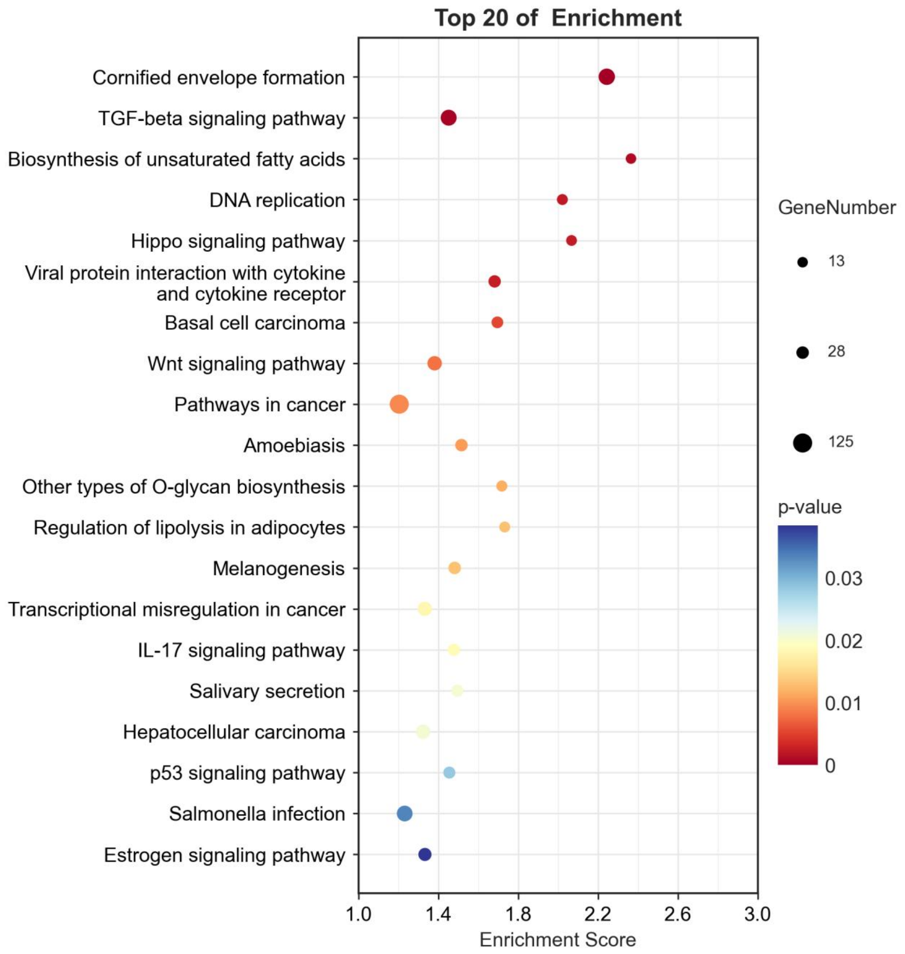

Figure S9 WGCNA for Module-Trait Relationships and dose-response curves of PTX upon INPPL1 overexpression and knockdown, related to Figure 6. (**A**) Topological analysis of the network showing scale independence and mean connectivity across soft thresholding powers, histogram of connectivity, and validation of scale-free topology at power = 28 ($R^2 = 0.8$, slope = -1.83). (**B**) Heatmap of the topological overlap matrix (TOM) with hierarchical clustering of gene modules. (**C**) Module-trait relationships of gene modules related to PTX. (**D**) Dose-response curves of PTX upon INPPL1 overexpression in three PTX-resistant organoids (ESC30-PDO, ESC34-PDO and ESC40-PDO). Data are from at least three independent experiments. Error bars represent means ± SD. ***, $P < 0.001$; EV, empty vector; OE, overexpression. (**E**) Dose-response curves of PTX upon INPPL1 knockdown in three PTX-sensitive organoids (ESC27-PDO, ESC31-PDO and ESC42-PDO). Data are from at least three independent experiments. Error bars represent means ± SD. ***, $P < 0.001$. NC, negative control; #, sh INPPL1.

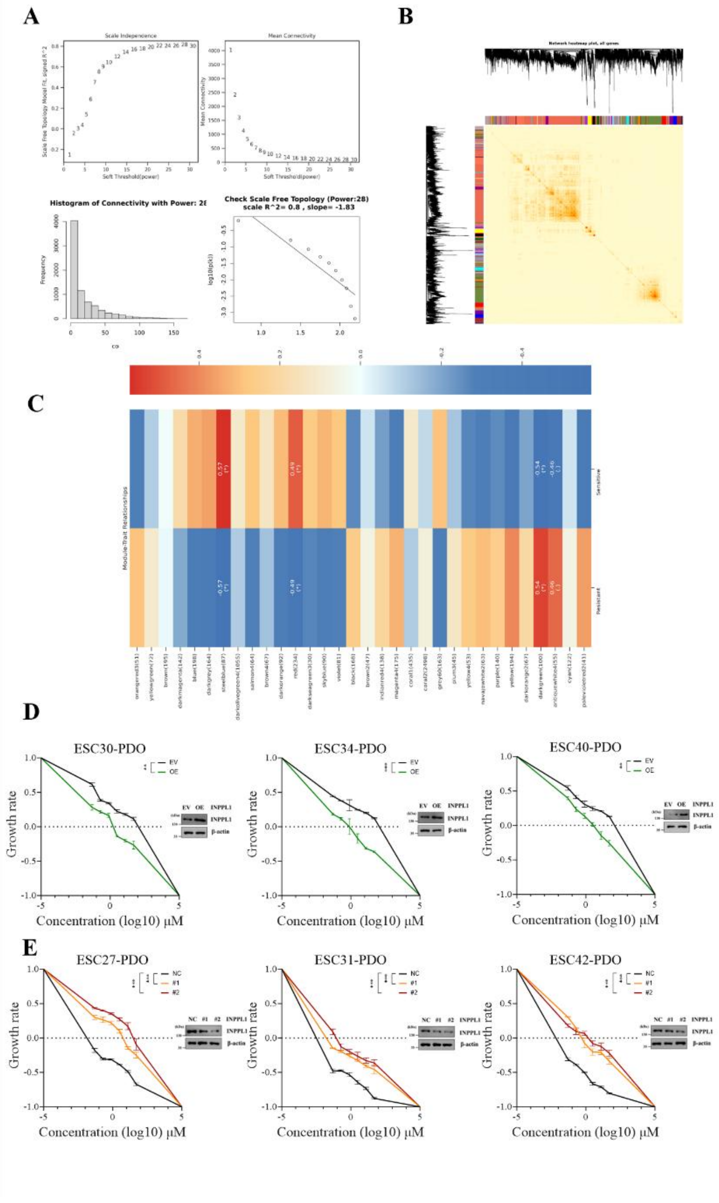

Table S7 Summary of expression changes of 129 genes and module correlation results, related to Figure 6.

Gene	log2FoldChange	p-value	q-value	Regulation	Modules	Moddule Membership
ACER2	-1.98	1.55E-06	1.57E-05	Down	red	0.72
ACTG1	-1.03	5.77E-06	5.01E-05	Down	red	0.65
ADAMTSL5	-1.85	4.89E-11	1.38E-09	Down	red	0.85
ALDH3B2	-2.16	0.00	0.00	Down	red	0.82
ANKRD50	-1.33	3.43E-06	3.15E-05	Down	red	0.78
ARHGAP27	-1.05	0.00	0.01	Down	red	0.85
ARHGAP32	-1.25	0.00	0.01	Down	red	0.87
ARHGEF10L	-2.08	0.00	0.00	Down	red	0.92
ARMCX2	-2.70	0.00	0.01	Down	red	0.59
ARRDC3	-1.22	0.01	0.02	Down	red	0.83
BATF2	-1.61	0.01	0.03	Down	red	0.93
BHLHE40	-1.84	5.24E-08	7.75E-07	Down	red	0.85
BNIPL	-3.23	7.95E-07	8.72E-06	Down	red	0.71
C15orf62	-2.53	1.72E-07	2.25E-06	Down	red	0.66
CA13	-2.06	0.02	0.05	Down	red	0.82
CAPN12	-1.25	0.01	0.03	Down	red	0.70
CCDC120	-1.31	0.00	0.00	Down	red	0.91
CCDC88B	-1.47	0.01	0.03	Down	steelblue	0.77
CCNG2	-1.72	5.90E-09	1.09E-07	Down	red	0.82
CDC42EP5	-3.82	4.76E-06	4.21E-05	Down	red	0.75
CDIP1	-1.11	0.00	0.00	Down	red	0.71
CSRNP1	-1.41	0.01	0.03	Down	red	0.72
CTSV	-1.63	0.01	0.02	Down	antiquewhite 4	0.70
CXCL2	-1.98	0.00	0.01	Down	red	0.86
DBI	-1.07	1.29E-06	1.34E-05	Down	antiquewhite 4	0.81
DENND2D	-1.29	0.00	0.00	Down	red	0.90
DOK4	-1.31	0.00	0.01	Down	red	0.75
DUOX1	-1.97	1.80E-12	6.72E-11	Down	red	0.80
DUOXA1	-2.29	3.31E-07	3.96E-06	Down	red	0.80
DUSP1	-1.57	0.00	0.02	Down	red	0.74
ECHDC3	-2.55	0.01	0.03	Down	red	0.74
EHBP1L1	-1.04	4.66E-08	6.99E-07	Down	red	0.60
EPHA1	-1.13	0.00	0.00	Down	red	0.74
EPHX3	-3.29	0.00	0.01	Down	red	0.90
EPIST	-3.22	0.00	0.01	Down	red	0.83
EVPL	-2.16	4.15E-12	1.45E-10	Down	red	0.73
FAAH	-1.26	0.02	0.04	Down	red	0.86
FBXO2	-1.24	0.00	0.01	Down	red	0.75
FHIP1A	-1.03	0.00	0.02	Down	red	0.58
FLNB	-1.08	1.95E-05	0.00	Down	red	0.55
FXYD5	-1.29	0.01	0.02	Down	red	0.75
GRHL1	-1.74	0.00	0.00	Down	red	0.84
H2AC6	-1.29	0.01	0.02	Down	red	0.58
HOXC6	-1.33	0.01	0.03	Down	red	0.63
HSBP1L1	-1.31	1.18E-05	9.40E-05	Down	red	0.70
IFNGR2	-1.02	1.84E-09	3.75E-08	Down	red	0.80
IL13RA1	-1.03	0.00	0.00	Down	red	0.72
IL17RE	-1.38	3.37E-06	3.10E-05	Down	red	0.86

Gene	log2FoldChange	p-value	q-value	Regulation	Modules	Moddule Membership
INPPL1	-2.40	0.01	0.03	Down	red	0.86
IRX5	-1.22	0.00	0.00	Down	red	0.64
ITGB7	-2.42	0.00	0.01	Down	red	0.87
JUNB	-1.09	0.01	0.02	Down	steelblue	0.55
KLF6	-1.18	0.01	0.02	Down	red	0.68
KLF8	-2.92	0.00	0.00	Down	red	0.88
KLHDC8B	-1.01	0.00	0.00	Down	red	0.63
KLK6	-4.09	8.14E-07	8.91E-06	Down	red	0.58
LDAF1	-1.25	1.12E-07	1.54E-06	Down	red	0.54
LEMD1	-2.11	0.00	0.00	Down	red	0.68
LEP	-4.55	0.00	0.01	Down	red	0.67
LIG4	-1.15	0.00	0.02	Down	red	0.60
LINC01451	-1.88	0.00	0.01	Down	steelblue	0.68
LINC02188	-2.94	0.01	0.03	Down	red	0.85
LINC02560	-1.52	0.00	0.02	Down	red	0.78
LINC02678	-1.41	0.01	0.03	Down	red	0.84
LOC100240735	-3.38	2.21E-05	0.00	Down	red	0.72
LOC105370964	-3.69	0.00	0.00	Down	red	0.86
LPAR5	-1.86	1.97E-07	2.53E-06	Down	red	0.67
LRAT	-2.44	0.00	0.00	Down	red	0.85
LRP10	-1.18	1.63E-06	1.63E-05	Down	red	0.61
LRRC75B	-1.05	0.00	0.01	Down	steelblue	0.64
LXN	-3.47	0.02	0.05	Down	red	0.48
MAST4	-1.32	0.00	0.00	Down	red	0.85
MGST3	-1.00	0.00	0.00	Down	antiquewhite 4	0.78
MINDY1	-1.31	0.00	0.01	Down	red	0.90
MMRN2	-1.58	8.25E-05	0.00	Down	red	0.43
MROH6	-1.68	1.29E-05	0.00	Down	red	0.57
MTSS1	-1.26	0.01	0.03	Down	red	0.57
MYD88	-1.37	0.00	0.00	Down	red	0.82
NAGK	-1.35	0.00	0.00	Down	red	0.64
NBEAL2	-1.59	2.79E-07	3.43E-06	Down	red	0.90
NLRX1	-1.30	7.72E-05	0.00	Down	red	0.83
NMU	-2.57	2.70E-08	4.26E-07	Down	red	0.78
NR1D1	-1.22	0.00	0.01	Down	red	0.44
NYNRIN	-1.92	0.02	0.04	Down	steelblue	0.79
PARVA	-1.36	1.17E-10	3.10E-09	Down	red	0.67
PCBP4	-1.12	0.00	0.01	Down	red	0.69
PITX1	-1.79	0.00	0.00	Down	red	0.87
PITX1-AS1	-1.51	3.62E-06	3.30E-05	Down	red	0.74
PLEKHG5	-1.41	1.96E-08	3.20E-07	Down	red	0.73
PLEKHM1	-1.16	3.36E-05	0.00	Down	red	0.72
PPP1R13L	-1.31	1.09E-06	1.15E-05	Down	red	0.74
PRICKLE4	-1.69	0.00	0.00	Down	red	0.88
PROM2	-1.47	0.02	0.05	Down	red	0.67
PRXL2B	-1.48	1.95E-07	2.50E-06	Down	red	0.71
QSOX1	-1.35	2.87E-05	0.00	Down	red	0.65
RAPGEF3	-2.02	0.00	0.01	Down	red	0.82
RAPGEFL1	-1.36	1.06E-06	1.12E-05	Down	red	0.76
REC8	-3.53	8.05E-07	8.82E-06	Down	red	0.66
RETSAT	-1.01	1.05E-07	1.45E-06	Down	red	0.57
RIPK4	-1.53	5.83E-05	0.00	Down	red	0.80
RPH3AL	-2.14	0.00	0.00	Down	red	0.70

Gene	log2FoldChange	p-value	q-value	Regulation	Modules	Moddule Membership
S100A3	-1.92	1.70E-07	2.23E-06	Down	red	0.70
SH3BP2	-1.26	5.66E-05	0.00	Down	steelblue	0.67
SIAE	-1.36	0.00	0.01	Down	red	0.75
SIM2	-3.02	7.46E-06	6.26E-05	Down	red	0.79
SLC22A15	-1.67	0.00	0.00	Down	red	0.86
SLC43A3	-1.90	0.01	0.02	Down	red	0.68
SNHG18	-3.31	0.00	0.01	Down	red	0.73
STXBP5	-1.37	0.00	0.00	Down	red	0.69
SYT8	-2.97	2.55E-06	2.42E-05	Down	red	0.78
SYTL1	-1.24	8.83E-05	0.00	Down	red	0.80
TACSTD2	-1.96	1.76E-06	1.75E-05	Down	red	0.74
TMEM184A	-1.77	0.00	0.00	Down	red	0.59
TMEM79	-2.21	4.79E-06	4.23E-05	Down	red	0.81
TNFRSF10A	-1.21	3.79E-08	5.81E-07	Down	red	0.63
TNFRSF10B	-1.13	1.87E-05	0.00	Down	red	0.75
TNFRSF14	-2.31	0.00	0.00	Down	red	0.85
TNNI2	-2.42	0.00	0.01	Down	red	0.72
TOB1	-1.05	0.00	0.00	Down	red	0.80
TOR4A	-2.04	0.00	0.00	Down	red	0.64
TRIM5	-1.09	0.01	0.02	Down	red	0.86
UBE2E1	-1.00	2.51E-05	0.00	Down	red	0.62
UNC13D	-2.10	0.00	0.01	Down	red	0.77
VAMP5	-1.95	0.00	0.00	Down	red	0.76
VSIR	-1.88	3.71E-07	4.39E-06	Down	red	0.87
YPEL3	-1.04	0.00	0.01	Down	red	0.73
ZDHHC21	-1.24	0.00	0.01	Down	red	0.53
ZNF750	-1.87	0.01	0.03	Down	red	0.89
ZNF888	-1.27	0.01	0.02	Down	antiquewhite 4	0.60

Figure S10 The results of ESC30-PDOX drug sensitivity assay, related to Figure 7. (**A**) Tumor volume changes over time in the ESC30-PDOX model treated with negative control, TP treatment group, and Doxorubicin group (n = 5, Mean ± SD). (**B**) Tumor images at the time of sacrifice. (**C**) Tumor weight at the time of sacrifice (n = 5, Mean ± SD). (**D**) Representative Ki-67 immunohistochemistry images and TUNEL immunofluorescence images. Scale bars are 100 µm. (**E**) Quantification of Ki-67 immunohistochemistry staining. (**F**) Quantification of TUNEL immunofluorescence staining.

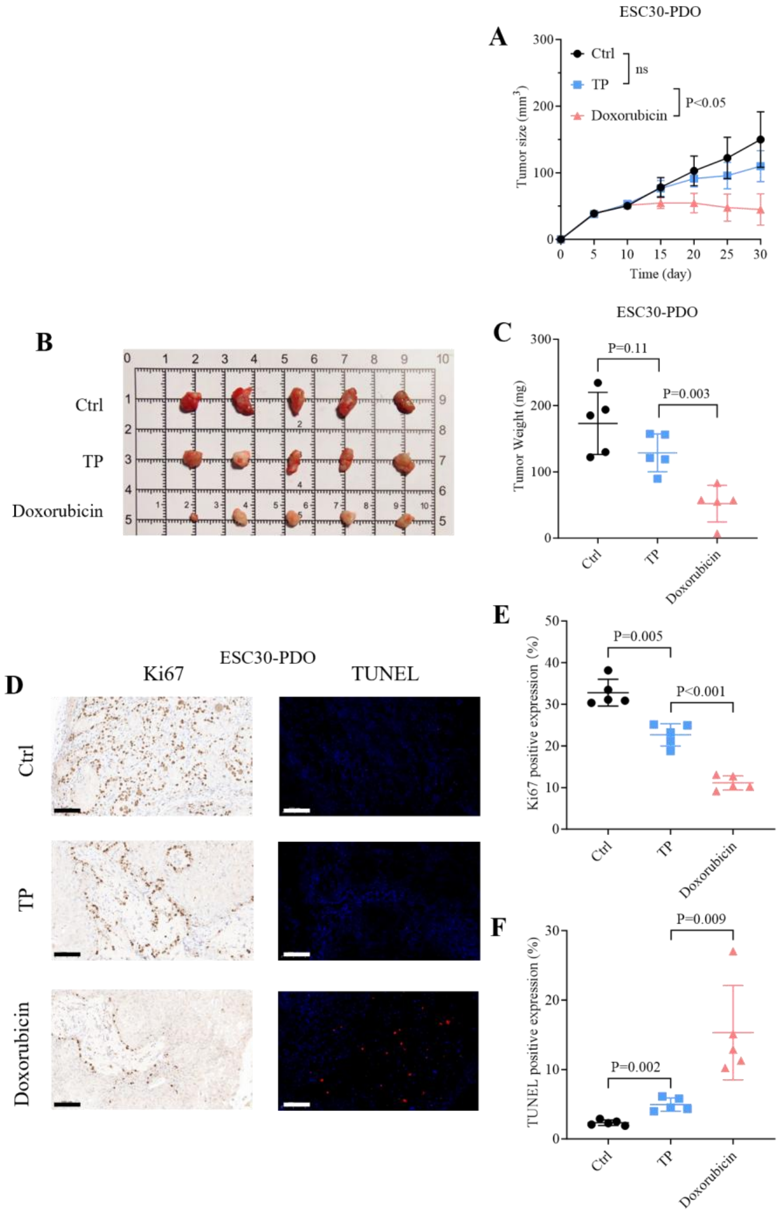

Figure S11 Representative histological images of subcutaneous xenograft tumors in the ESC22-PDO and ESC30-PDOX models, related to Figure 7. Scale bars are 200 μm.

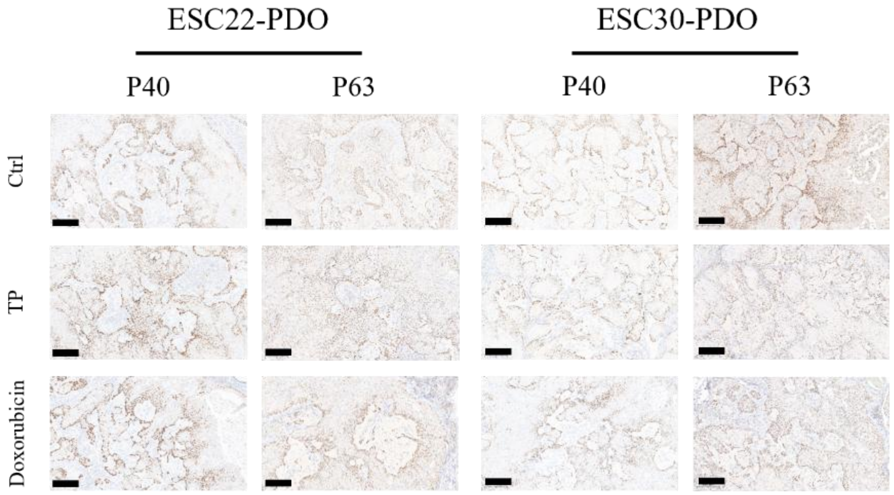

Figure S12 Dose–response curves for paclitaxel and cisplatin in A549 and drug-resistant A549/DDP and A549/Tax cell lines. (A) Dose-response curves of different cell lines treated with Paclitaxel. (**B**) Dose-response curves of different cell lines treated with Cisplatin.

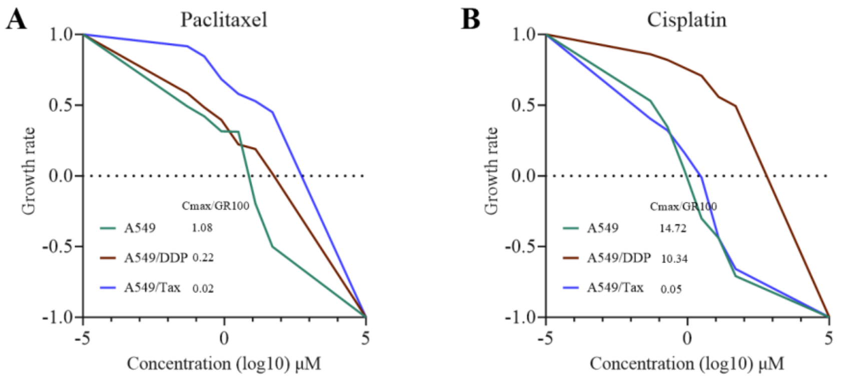

Supplement: Supplementary file 3 — Document S1. Figures S1–S12 and Tables S3–S7 [file CTM2-15-e70534-s002.pdf]
